# Advances in Drug Delivery Science for Diacerein: Strategies to Enhance Solubility, Bioavailability, and Pharmacokinetic Performance

**DOI:** 10.3390/pharmaceutics17121539

**Published:** 2025-11-29

**Authors:** Maryam Naseri, Sajjad Ghobakhlou, Niloofar Heidarizade, Mohammad Emad Akbari, Alireza Lotfabadi, Soroor Sadegh Malvajerd, Zhila Izadi, Hassan Maleki

**Affiliations:** 1 Student Research Committee, Kermanshah University of Medical Sciences, Kermanshah 6715847141, Iran; 2 Pharmaceutical Sciences Research Center, Health Institute, Kermanshah University of Medical Sciences, Kermanshah 6715847141, Iran; 3 USERN Office, Kermanshah University of Medical Sciences, Kermanshah 6715847141, Iran; 4 Nano Drug Delivery Research Center, Health Technology Institute, Kermanshah University of Medical Sciences, Kermanshah 6715847141, Iran

**Keywords:** anti-inflammatory, bioavailability, diacerein, nanocarrier, osteoarthritis, solubility

## Abstract

Diacerein is known as a disease-modifying anti-inflammatory drug, primarily used for the treatment of osteoarthritis. Despite its therapeutic potential, the clinical use of diacerein is hindered by poor aqueous solubility, low bioavailability, liver issues, and gastrointestinal side effects, particularly diarrhea. To address these limitations, various innovative pharmaceutical formulation approaches have been explored, including physical modifications, chemical complexation, nanotechnology-based drug delivery systems, and synergistic combination therapies. This review highlights progress in formulation approaches aimed at enhancing the solubility and therapeutic profile of diacerein. Special emphasis is placed on lipid-based carriers, vesicular systems, pH-responsive hydrogels, and dissolving microneedles. Together, these strategies provide a comprehensive platform for the rational design of diacerein formulations, offering promising avenues to overcome its clinical limitations and improve patient outcomes. The insights presented here may also guide the development of more effective delivery systems for other poorly soluble anti-inflammatory agents.

## 1. Introduction

Diacerein (DCN) is a promising anti-inflammatory agent with disease-modifying potential, which makes it beneficial for treating a spectrum of inflammatory conditions, including osteoarthritis (OA), epidermolysis bullosa (EB), psoriasis, diabetic complications, and ocular inflammatory diseases [1,2,3,4]. At the outset, due to its anti-inflammatory, chondroprotective, and analgesic properties, it was prescribed for the management of joint diseases. Subsequently, the therapeutic potential of DCN has positioned it as a key agent in the management of other inflammatory diseases [1,2,5,6,7,8]. DCN selectively inhibits interleukin-1 beta (IL-1β) and tumor necrosis factor-alpha (TNF-α), thereby avoiding regular adverse effects associated with NSAIDs, such as gastrointestinal (GI) toxicity and cardiovascular complications. This mechanism enables diacerein to serve as a safer option for managing long-term inflammatory diseases [9,10].

Despite such therapeutic advantages, DCN faces significant pharmacokinetic challenges, including poor aqueous solubility, extensive first-pass metabolism, and low bioavailability [11,12,13], all of which limit its clinical effectiveness [14,15,16].

To overcome these limitations, researchers have explored a range of innovative oral formulation strategies designed to enhance DCN’s bioavailability. Among these, nanoparticle-based formulations, lipid-based carriers, and solid dispersions (SDs) have demonstrated significant promise [16,17,18]. Cyclodextrin (CD) inclusion complexes improve drug stability and solubility by forming host–guest molecular structures that protect against premature degradation [7]. Solid dispersion technology involves dispersing DCN within hydrophilic polymer matrices, thereby increasing dissolution rate and promoting more consistent pharmacokinetic profiles [19,20].

Beyond oral formulations, alternative drug delivery approaches, such as intra-articular injections and transdermal systems, have been developed to circumvent GI side effects and improve localized drug release. Liposomal, hyalurosomal, and niosomal formulations facilitate targeted site delivery, thereby reducing systemic exposure and improving drug retention at the site of inflammation [8,19,20]. Furthermore, transdermal nanoemulgel systems and microemulsions offer enhanced skin penetration and controlled drug release, making them promising options for psoriasis and other inflammatory skin conditions [21,22].

Despite these advancements, challenges remain in achieving complete optimization of diacerein’s pharmacokinetics. Current formulation strategies predominantly focus on overcoming these challenges. Additionally, the majority of studies emphasize drug delivery mechanisms [15,23,24,25], often overlooking the potential of physicochemical modifications to improve DCN’s stability and systemic retention further.

Importantly, the relevance of selecting DCN is supported by its emerging multi-target pharmacological profile. Recent evidence, including a comprehensive review by Almezgagi et al., highlights DCN’s anti-inflammatory, antioxidant, anti-fibrotic, and neuroprotective effects across diverse conditions, mediated via the IL-1β, TNF-α, NF-κB, and MAPK/ERK pathways. These properties, coupled with its generally acceptable safety profile at optimized doses, make DCN a strong candidate for advancement through modern drug-delivery and physicochemical optimization strategies [26]. Furthermore, early-phase clinical investigations in osteoarthritis, metabolic inflammation, and type 2 diabetes suggest potential therapeutic expansion, reinforcing the scientific justification for a renewed evaluation of DCN’s formulation landscape [27].

This review aims to provide an integrated evaluation of recent advancements in diacerein formulation, combining both physicochemical modification strategies and nano-/advanced drug-delivery approaches to present a cohesive and comprehensive understanding of the current formulation landscape. Although the findings of current studies may offer limited insight into fully addressing these challenges, efforts have been made to scrutinize and analyze the available data. Therefore, this study provides a comprehensive framework for optimizing DCN’s pharmacokinetic profile and long-term therapeutic efficacy across multiple inflammatory disease conditions.

## 2. Absorption of Diacerein: Role of Physicochemical Properties

DCN, an anthraquinone derivative (1,8-diacetoxy-3-carboxyanthraquinone) [9], falls under the Biopharmaceutics Classification System (BCS) Class II. DCN is characterized by very low aqueous solubility (~7–10 µg/mL, depending on pH) [1,3,28] and a log *p* value of approximately 2.4 based on experimental patent data (EP2349289B1) [29]. These physicochemical constraints limit its dissolution and absorption in physiological fluids, despite its high membrane permeability. With a molecular weight of 368.29 g/mol and high lipophilicity, DCN demonstrates limited solubility and slow dissolution rates [10,15]. The dissolution rate of DCN represents the principal rate-limiting step in its pharmacokinetics, contributing to a relatively low oral bioavailability of 35–56% [10,13,14,30,31]. Moreover, DCN undergoes pH-dependent hydrolysis under acidic conditions (pH ~1.2), which further reduces bioavailability and increases pharmacokinetic variability. Conversely, solubility improves markedly at intestinal pH (~6.8, neutral to slightly alkaline), highlighting the need for modified-release formulations, such as co-crystallization, solid dispersion, and complexation techniques, to enhance absorption and prevent premature degradation [10,30,32].

**Figure 1:**
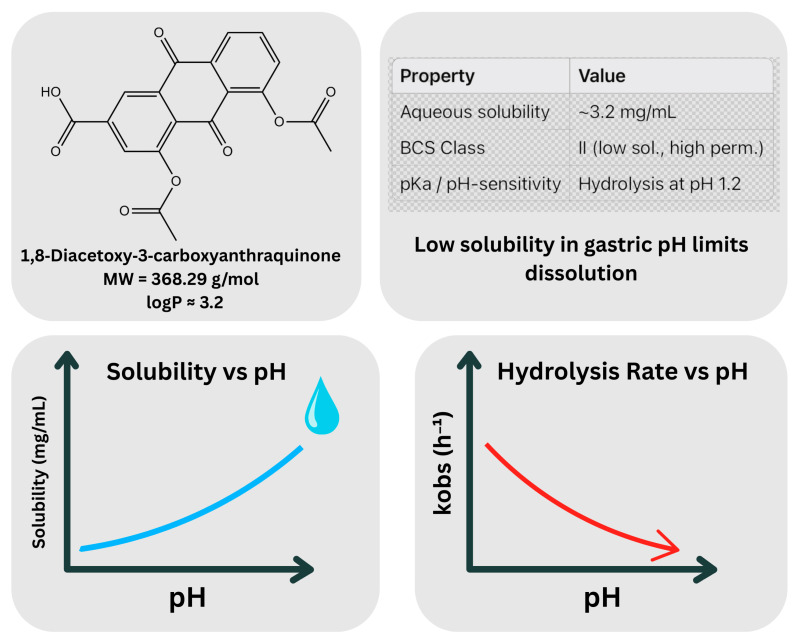
Key physicochemical features of diacerein, illustrating its chemical structure, low intrinsic solubility, BCS Class II status, and pH-dependent behavior in both solubility and hydrolytic degradation.

These physicochemical limitations and their pH-dependent behavior are illustrated in Figure 1, which shows the chemical structure of DCN along with its physicochemical profiles.

Following oral administration, DCN is rapidly hydrolyzed in the intestine to rhein, its active metabolite, which exhibits a mean elimination half-life of approximately 7–8 h under steady-state conditions [33]. This active metabolite is responsible for its anti-inflammatory and chondroprotective effects [10,26]. However, rhein undergoes extensive first-pass hepatic metabolism, leading to its rapid conjugation into glucuronide and sulfate derivatives that are excreted renally, thereby significantly reducing systemic drug availability [17,30,34,35]. Peak plasma concentrations are typically reached within 2–3 h, but bioavailability remains inconsistent due to high inter-individual variability in metabolism [10,32].

Molecular modeling studies suggest that DCN’s poor solubility is primarily attributed to its rigid crystalline structure and strong intermolecular hydrogen bonding, both of which significantly impede its dissolution in biological fluids [10,11,36]. In addition, its high hydrophobicity further limits systemic absorption, reinforcing the need for effective solubility-enhancing strategies [15,26,30,35,37].

**Figure 2:**
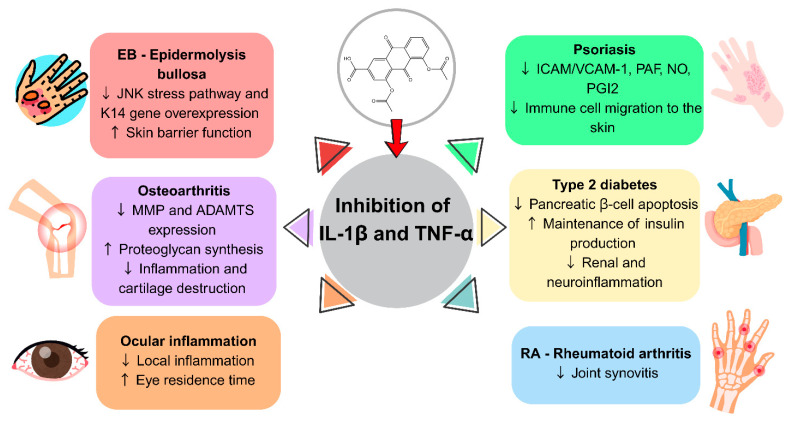
Expanded therapeutic spectrum of diacerein across inflammatory and degenerative disorders. The figure illustrates how IL-1β and TNF-α inhibition drives downstream disease-modifying effects, including reduced matrix degradation in osteoarthritis, suppression of JNK-mediated stress responses in epidermolysis bullosa, modulation of immune cell migration in psoriasis, preservation of pancreatic β-cell function in type 2 diabetes, attenuation of joint synovitis in rheumatoid arthritis, and decreased local inflammation with prolonged residence time in ocular diseases.

Compared to other pharmacological treatments for OA, DCN exhibits distinct pharmacokinetic and pharmacodynamic profiles, especially in contrast to NSAIDs and symptomatic slow-acting drugs (SYSADOAs). NSAIDs, such as ibuprofen, diclofenac, and celecoxib, act rapidly by inhibiting cyclooxygenase (COX) enzymes and reducing prostaglandin synthesis, thereby providing immediate symptom relief [35]. These agents typically demonstrate higher aqueous solubility, predictable oral absorption, and rapid systemic activity; however, they are associated with gastrointestinal and cardiovascular risks [14,35]. While NSAIDs offer rapid symptom relief, their long-term safety remains a concern. In contrast, DCN acts by inhibiting IL-1β and TNF-α, resulting in a slower onset of action but with potential for disease modification. This mechanism may delay cartilage degradation and slow the progression of OA, thereby providing long-term chondroprotective benefits [10,30]. The therapeutic effects of diacerein are primarily mediated through the inhibition of IL-1β and TNF-α pathways, which contribute to a broad spectrum of inflammatory and degenerative diseases. These disease-modifying effects are summarized in Figure 2.

Compared to SYSADOAs such as glucosamine and chondroitin sulfate, DCN exhibits superior anti-inflammatory activity, primarily due to its ability to inhibit IL-1β, a key cytokine involved in OA progression [10,35]. Despite these advantages, challenges related to DCN, discussed more extensively in the following section, necessitate formulation modifications to achieve consistent and effective therapeutic outcomes [30].

## 3. Factors Limiting the Bioavailability and Stability of DCN

As mentioned before, a significant obstacle to DCN’s clinical use is its physicochemical properties, which result in delayed dissolution and slow onset of action, thereby limiting its effectiveness in managing inflammatory diseases [38,39,40,41,42]. This challenge also hinders pharmaceutical development, contributing to inconsistent patient responses, safety concerns, raised costs, and heightened toxicity risks [40,43]. This limitation restricts DCN’s dissolution in GI fluids, a critical step for oral drug absorption [44]. Furthermore, the crystalline form of DCN reduces its solubility, as the strong lattice structure of crystalline Diacerein hinders molecular diffusion, resulting in slower dissolution compared to its amorphous form [45]. Likewise, low and unpredictable absorption of DCN may increase the risk of side effects, such as stomach irritation, soft stools, and urine discoloration, as patients often require higher doses to achieve the desired therapeutic effects [12,46,47]. According to a Cochrane systematic review of seven randomized controlled trials involving 1462 participants with osteoarthritis, patients treated with diacerein for 2 to 36 months experienced a significantly higher incidence of adverse effects compared to those receiving a placebo. The main side effect was diarrhea, with a relative risk of 3.52 and an absolute risk increase of 24%, implying that one additional adverse reaction occurs for every four patients treated with DCN [12]. Consequently, due to these clinical challenges, the oral use of DCN-containing medicines in patients aged 60 years or older with rheumatoid arthritis (RA) or OA has been restricted [32,33].

Another major pharmacokinetic limitation is the rapid enzymatic hydrolysis of DCN in the GI tract, where it is extensively converted into rhein before reaching systemic circulation, leading to low bioavailability and reduced therapeutic efficacy [38,40,41,48,49]. The metabolic instability also contributes to significant GI irritation and diarrhea, typical side effects that compromise long-term patient adherence [38,40,41,50]. Additionally, first-pass metabolism and enzymatic degradation further diminish systemic drug retention, highlighting the need for controlled-release and targeted formulations to enhance pharmacokinetic stability [24,42,51]. Given its short biological half-life, DCN necessitates frequent dosing, resulting in plasma level fluctuations and inconsistent therapeutic effects, which further complicates treatment regimens for chronic inflammatory conditions [3,4,42,50].

Beyond bioavailability concerns, DCN poses significant formulation challenges due to its instability in aqueous solutions, undergoing rapid degradation in both acidic and alkaline environments, which further compromises solubility and systemic absorption [23,52,53]. Moreover, in certain topical and transdermal applications, disease-related alterations in skin integrity—such as those observed in psoriasis or inflammatory conditions—may reduce the effective diffusion of DCN across biological membranes, leading to variability in systemic absorption and therapeutic response [1,21,48,52,54,55,56,57,58].

Similarly, DCN’s limited intra-articular retention reduces localized drug availability, underscoring the importance of biodegradable, sustained-release carriers for targeted treatment in inflammatory conditions [55,59]. In addition, its instability in conventional topical formulations, manifested through burst drug release and precipitation, undermines the effectiveness of alternative drug delivery systems [60,61,62]. The crystalline nature of DCN also impairs dissolution rates and systemic absorption, contributing to fluctuations in plasma drug levels and variability in therapeutic response, thereby necessitating optimized formulation strategies [20,43,63]. From a pharmaceutical and regulatory standpoint, the lack of standardized quality control methods for DCN, particularly in multi-drug formulations, presents challenges in analytical procedures, impurity detection, and stability assessment, emphasizing the importance of robust monitoring techniques [22,64].

## 4. Enhancing Bioavailability of DCN: Physical to Nanocarrier-Based Approaches

Given the limitations of DCN’s rapid enzymatic hydrolysis, and low systemic retention, numerous formulation strategies have been explored to enhance its therapeutic efficacy. These strategies can be broadly categorized into physicochemical modifications, chemical approaches, and nanotechnology-based drug delivery systems, each offering unique mechanisms to optimize dissolution, absorption, and sustained drug release. The distinct advantages and limitations of examples of each method are summarized in Table 1.

Physicochemical modifications aim to enhance the dissolution rates and absorption of DCN by altering its physical characteristics [65,66,67,68]. Techniques such as particle size reduction through micronization, nanocrystal formation, and spray drying enable an increase in surface area, thereby improving dissolution and oral bioavailability. Amorphization, which disrupts the drug’s crystalline structure, further enhances wettability and dissolution kinetics, resulting in improved bioavailability [23,53]. Additionally, co-processing with bio-enhancers and hydrophilic carriers—such as cyclodextrin inclusion complexes, liposomal and proliposomal systems, solid dispersions, and topical emulsifying formulations—has been reported to improve the solubility, permeability, and dissolution rate of Diacerein, resulting in enhanced bioavailability [19,40,53,69,70].

Alternatively, chemical modifications aim to modify DCN’s molecular properties to enhance systemic bioavailability [66,71,72,73]. Additionally, CD complexation and co-crystal formation enhance dissolution rates and facilitate controlled drug release, enabling more predictable pharmacokinetics and prolonged therapeutic effects [4,74].

Nanotechnology-based drug delivery systems offer targeted and controlled-release formulations that enhance bioavailability while minimizing side effects [43,46,71,75]. Nanocarriers such as liposomes, micelles, and nanoemulsions improve both oral and transdermal bioavailability and reduce GI irritation [23,53]. Biodegradable polymer-based carriers facilitate sustained intra-articular drug release, improving localized treatment for inflammatory conditions [60,76]. Dissolvable microneedles provide a minimally invasive approach for transdermal delivery, enabling controlled drug release and improving patient compliance [20,53]. Furthermore, emulsification-based systems, including self-emulsifying drug delivery systems (SEDDS) and nanoemulsions, enhance the dissolution, and absorption of Diacerein, leading to improved bioavailability [53].

In EB, nanoemulgels and nanofibrous dressings may enhance drug penetration and enable controlled release, thereby promoting wound healing and patient comfort. In psoriasis, liposomal and polymeric nanoformulations may stabilize DCN, providing longer-lasting therapeutic effects [77,78]. In ocular inflammation, mucoadhesive nanoparticles and liposomal eye drops can enhance drug retention and absorption, promising them for the treatment of uveitis and dry eye syndrome [79,80]. For diabetes, DCN’s anti-inflammatory properties aid in managing chronic inflammation, nephropathy, and neuropathy; however, low bioavailability limits its therapeutic efficacy [81]. Nanocarriers and enteric-coated formulations may enhance systemic retention, while smart hydrogels and nanofiber dressings show promise in improving diabetic wound healing [82,83].

All of the mentioned strategies offer promising pathways to overcome DCN’s pharmacokinetic limitations, ensuring improved absorption, sustained release, and enhanced clinical outcomes. Nevertheless, selecting the most appropriate approach depends on the drug’s physicochemical properties and the intended delivery route. Future direction may focus on hybrid systems that integrate efficacy, stability, and safety. In the following sections explore these key strategies and their impact on enhancing DCN’s therapeutic performance.

**Table 1:** Comparative analysis of solubility enhancement techniques for DCN.

Category	Technique	Mechanism/Description	Advantages	Limitations	Ref.
Physical Modification Approaches	Micronization	Reducing the particle size of Diacerein API to increase surface area and improve wetting; micronized DCN shows significantly faster dissolution compared with unmicronized DCN.	Enhances dissolution rate; improves capsule performance; reduces variability in drug release	Does not substantially change intrinsic solubility; may require careful control of flow properties	[65,66,67,84]
	Nanonization	Reduction in DCN into nanoscale particles via nano-carrier engineering (e.g., lecithin–gold hybrid nanocarriers) to increase surface area and dissolution kinetics.	Enhances saturation solubility and improves oral bioavailability	Requires stabilizers; risk of aggregation and complexity in formulation	[85]
	Solid dispersion/Amorphization	Diacerein dispersed in hydrophilic polymers (e.g., PVP K30; PEG; Soluplus) via kneading or solvent evaporation, leading to reduced crystallinity and partial/complete amorphization.	Markedly improves dissolution rate, enhances solubility, increases oral bioavailability (up to 2–3 fold).	Potential physical instability due to amorphous transitions; polymer ratio-dependent performance.	[20,40,86]
Chemical Modification Approaches	Prodrug (Mutual/Co-drug)	Covalent conjugation of Diacerein with antioxidant molecules (e.g., thymol) to mask the acidic group, improve lipophilicity, reduce gastric irritation, and enhance oral absorption.	Improved lipophilicity, enhanced absorption, reduced ulcerogenicity, controlled release, improved anti-arthritic activity.	Requires enzymatic cleavage; additional synthesis steps; stability profiling needed.	[87]
Physicochemical Modifications	Co-crystals	Formation of new crystalline phases with coformers (e.g., urea, tartaric acid, isonicotinamide, nicotinamide, theophylline, β-resorcylic acid) resulting in modified lattice structure and improved molecular interactions	Significantly enhances aqueous solubility, intrinsic dissolution rate, and biopharmaceutical performance; improves tabletability and physicomechanical properties	Requires selection of suitable coformers; solid-state characterization is essential; potential stability concerns depending on coformer	[74,88,89]
	Cyclodextrin Complexation	Formation of 1:1 inclusion complexes of Diacerein with β-CD or HP-β-CD via kneading, co-evaporation, or freeze-drying; DCN molecules become partially or fully entrapped inside the hydrophobic CD cavity, increasing amorphousness and reducing hydrolysis rate	Markedly improves aqueous solubility, dissolution rate, and apparent bioavailability; stabilizes DCN by decreasing hydrolysis rate; enhances tabletability and enables fast-disintegrating formulations	Limited drug loading; decreased complexation efficiency at higher CD concentrations; CD amounts may increase bulk of dosage form	[4,90,91]
Advanced Formulations	SMEDDS/SNEDDS (Self-micro/nano-emulsifying lipid-based systems)	Spontaneous formation of micro/nano-sized emulsions upon aqueous dilution, enhancing solubilization and dissolution of Diacerein.	Significantly increases solubility, dissolution rate, and oral bioavailability.	High surfactant load may cause GI irritation; limited drug loading capacity.	[92,93]
	Nanosuspensions	Submicron crystalline particles of diacerein prepared by precipitation, high-shear homogenization, or media milling, stabilized with polymers/surfactants to prevent aggregation	Significant increase in saturation solubility, rapid dissolution, improved permeability, higher oral bioavailability, and potential reduction in diarrheal side effects (especially with chitosan-coated nanocrystals)	Requires stabilizers; risk of aggregation; possible Ostwald ripening; lyophilization needed for stability	[49,94]
	Polymeric Nanoparticles	Diacerein encapsulated in biodegradable polymers (e.g., chitosan–chondroitin sulfate, PLGA) forming nano-sized carriers that enable sustained release, improved stability, and enhanced tissue penetration	Sustained release up to several days; reduced gastrointestinal side effects; improved anti-inflammatory effect; targeted intra-articular or transdermal delivery	Complex preparation process; need for optimization of polymer ratios; potential variability in entrapment efficiency	[95,96]
	Lipid-based Systems	Lipid nanoparticles such as solid lipid nanoparticles (SLNs) and nanostructured lipid carriers (NLCs) use solid or mixed lipid matrices to incorporate Diacerein, enhancing solubilization, promoting lymphatic uptake, and enabling sustained topical or oral delivery.	Improve bioavailability (2.7–3.7-fold), reduce gastrointestinal side effects, provide sustained and controlled release, improve topical permeation, and protect the drug in amorphous or molecularly dispersed form.	Potential drug leakage during storage, stability concerns, scale-up complexity, need for optimized lipid/surfactant ratios.	[15,97,98]

Abbreviations: API: Active Pharmaceutical Ingredient, CD: Cyclodextrin, GI: Gastrointestinal, NLCs: Nanostructured Lipid Carriers, PLGA: Poly(lactic-co-glycolic acid), PVP: Polyvinylpyrrolidone, PEG: Polyethylene Glycol, SMEDDS: Self-Microemulsifying Drug Delivery System, SNEDDS: Self-Nanoemulsifying Drug Delivery System, SLNs: Solid Lipid Nanoparticles.

### 4.1. Physical Strategies to Improve DCN Solubility

Various physical formulation strategies have been developed, including SD, fast-dissolving tablets (FDTs), matrix-based controlled-release tablets, microsphere-based formulations, and gastro-retentive (GR) systems For DCN. These approaches aim to enhance dissolution, improve absorption, and prolong therapeutic effects, thereby increasing efficacy and patient compliance [65,66,67,73,99]. A comparative analysis of these strategies with more detail is presented in Table 2.

#### 4.1.1. Solid Dispersion System for Enhanced Dissolution

Solid dispersion (SD) is a widely studied technique for enhancing the solubility of poorly water-soluble drugs by dispersing them in hydrophilic carriers. This method reduces crystallinity, increases wettability, and improves dissolution characteristics. Various SD formulations have been developed for DCN, including Pluronic^®^ F127-based SDs, which increased the dissolution rate by 8.6-fold compared to crystalline DCN [6], and ternary SDs incorporating Pluronic^®^ F127, Solutol^®^ HS15, and PEG 35K, which improved solubility to 70.2 µg/mL and achieved 79.28% drug release within 60 min [3]. PEG 8000 and polyvinyl pyrrolidone (PVP) K30-based SDs demonstrated a 10.83-fold increase in dissolution efficiency and a 6.07-fold reduction in mean dissolution time (MDT) [40]. In contrast, mannitol-based SDs (1:5 drug-to-carrier ratio) exhibited optimal dissolution performance due to improved wettability and hydrogen bonding [100]. In addition, as shown in Table 2, Pluronic^®^ F68-based SDs for immediate release could achieve 4.04-fold higher dissolution efficiency and a 6.6-fold faster release rate [101], whereas PVP K30-based SDs could reach 95.87–100% dissolution, providing substantial improvement in bioavailability [20].

#### 4.1.2. Fast-Dissolving Tablets for Rapid Drug Absorption

Fast-dissolving tablets (FDTs), which rapidly disintegrate in the mouth, offer a convenient approach for immediate drug absorption without the need for water, particularly beneficial for elderly patients who are more susceptible to DCN’s GI side effects. While FDTs provide rapid disintegration and enhanced ease of administration, the improved dissolution and bioavailability reported in the literature are influenced not only by the FDT platform but also by the solubility-enhancing excipients incorporated into these formulations [39,101,102]. As reported in an investigation, a formulation of Pluronic^®^ F68-based FDTs exhibited a 4.04-fold increase in dissolution efficiency and a 6.6-fold faster drug release [101]. While PEG 8000 and Sorbitol-based FDTs enhanced bioavailability by 2.66-fold compared to commercial DCN capsules and also reduced gastrointestinal irritation—effects that may be partially attributed to the solubilizing action of PEG 8000 [39]. Additionally, FDTs formulated with Crospovidone and Camphor achieved rapid disintegration and followed first-order drug release kinetics, making them particularly suitable for patients with swallowing difficulties [102]. However, the observed benefits of the mentioned FDT delivery systems might arise from a combination of rapid disintegration and the physicochemical properties of incorporated excipients.

#### 4.1.3. Immediate-Release and Gastro-Retentive Formulations

Immediate-release (IR) and gastro-retentive (GR) formulations are designed to modify drug dissolution and absorption kinetics, thereby optimizing systemic exposure. Mandawgade et al. investigated an IR formulation of DCN using microparticle-based sugar spheres to increase surface area compared to the commercial capsule Art^®^50. They reported that the IR formulation achieved 90% drug release within one hour, significantly higher than Art^®^50 (75%), leading to a 1.7-fold increase in the area under the curve from 0–6 h (AUC_0–6h_) in both in vitro and in vivo evaluations [103]. Conversely, the GR formulation, which incorporated a floating polymer blend to prolong gastric retention and slow the conversion of Diacerein to rhein, achieved 96% drug release within two hours and a 1.2-fold increase in AUC_0–6h_. This slower and more controlled release profile resulted in a reduced and attenuated systemic rhein peak compared with the commercial product, thereby lowering the likelihood of sharp gastrointestinal exposure, which is strongly associated with DCN-induced diarrhea. Clinical data further confirmed that compared with Art^®^50, the IR and GR formulations produced 1.7-fold and 1.2-fold increases in AUC_0–6h_, respectively, while the GR system generated a smoother plasma profile with lower maximum rhein concentrations [101], a factor that may contribute to reduced GI side effects [103].

#### 4.1.4. Matrix-Based Controlled-Release Tablets for Prolonged Action

Matrix-based controlled-release formulations regulate drug release kinetics, maintaining steady plasma concentrations and reducing dosing frequency. Kabir et al. reported that Methocel K100M and K4M-based matrix tablets sustained drug release for up to eight hours, thereby improving patient compliance [104]. Furthermore, the optimized asymmetric osmotic pump tablets (Opadry^®^ CA-coated) provided a controlled and highly linear release pattern over a 24-h period, consistent with zero-order kinetics (R^2^ = 0.977). The formulation demonstrated an initial release phase influenced by the incorporated ternary solid dispersion—showing approximately 79% drug dissolution within the first hour—followed by a sustained and uniform release profile throughout the remaining period, ultimately enhancing bioavailability and minimizing plasma concentration fluctuations [3].

#### 4.1.5. Microsphere-Based Formulations for Sustained Absorption

Microsphere-based drug delivery also provides controlled release, reducing GI side effects and ensuring prolonged therapeutic efficacy. DCN-loaded microspheres coated with ethyl cellulose and hydroxypropyl methylcellulose (HPMC) could increase the T_max_ from 2.67 h to 4 h, thereby extending systemic absorption [105]. Additionally, fluid-bed coated microspheres exhibited a prolonged T_max_ and controlled drug release, with an entrapment efficiency (EE) of 61% [105].

**Figure 3:**
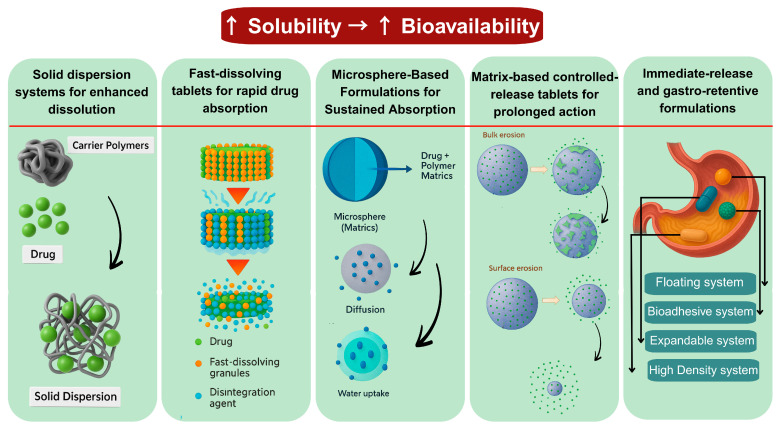
Physical modification strategies used to enhance diacerein’s solubility and oral bioavailability. The figure illustrates key platform categories, including solid dispersions that improve wettability and dissolution, fast-dissolving tablets designed for rapid disintegration and absorption, microsphere-based matrices enabling sustained release through gradual diffusion, matrix-controlled tablets that provide prolonged action via surface or bulk erosion, and gastro-retentive systems that extend gastric residence time to optimize dissolution and absorption.

Various physical strategies such as solid dispersions, fast-dissolving tablets, microspheres, and matrix-based systems have been developed to improve dissolution and absorption of DCN. These approaches are summarized in Figure 3.

### 4.2. Physicochemical Strategies for Enhancing DCN Solubility

**Figure 4:**
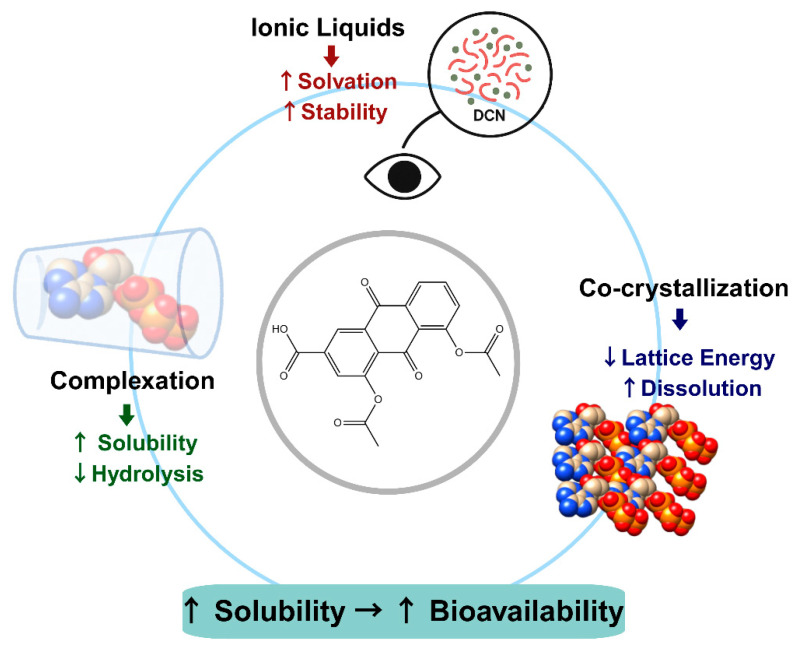
Key physicochemical strategies for improving diacerein performance, including cyclodextrin complexation, pharmaceutical co-crystallization, and ionic liquid-based systems, all aimed at enhancing solubility and overall bioavailability.

Physicochemical modifications, as well as complexation strategies, have been developed to enhance dissolution, modify crystallinity, and improve stability. These approaches aim to optimize drug absorption, stabilize DCN against hydrolysis, and modify drug release profiles for controlled or immediate action [4,7,62,74,106,107]. Representative strategies are illustrated in Figure 4. This section discusses these various modification techniques, their mechanisms of action, and their comparative effectiveness, as summarized in Table 3.

#### 4.2.1. Cyclodextrin Inclusion Complexation for Enhanced Solubility and Stability

Inclusion complexation, particularly with cyclodextrins (CDs), is a well-established technique to overcome limitations of hydrophobic drugs. CDs are cyclic glucose oligomers that form water-soluble complexes with a wide range of guest molecules, owing to their hydrophilic exterior and relatively hydrophobic cavity. Their biocompatibility, low toxicity, and non-immunogenic nature make them highly attractive carriers in pharmaceutical formulations [108].

Studies have shown that DCN can be effectively complexed with CDs, such as hydroxypropyl-β-cyclodextrin (HPβCD) and β-cyclodextrin (β-CD), resulting in significantly improved aqueous solubility and dissolution rates [4,7]. Both studies consistently demonstrated a 1:1 stoichiometric ratio between DCN and the respective cyclodextrins, confirming the formation of stable inclusion complexes [4,7]. Petralito et al. confirmed complex formation with HP-β-CD in both solution and solid-state through spectroscopic analyses, showing enhanced solubility and hydrolytic resistance [7]. DCN-HPβCD complexes were prepared using four methods: physical mixture, kneading, co-evaporation, and freeze-drying. Among these, freeze-drying produced the best results, achieving 100% drug dissolution in 5 min compared to only 55% in 60 min for pure DCN. Additionally, Characterization confirmed stable complex formation with reduced crystallinity, enhanced wettability, and 50% improvement in chemical stability [7]. Similarly, another study reported that DCN–β-CD and DCN–HPβCD complexes also followed a 1:1 host–guest stoichiometry, with HPβCD-based complexes increasing solubility to 4.36 mg/mL—substantially higher than the 3.197 mg/L of unmodified DCN—and achieving 99.95% release within 60 min [4]. These findings support CD inclusion as a promising approach for fast-disintegrating oral formulations.

Phase solubility studies have demonstrated that both β-CD and HPβCD form stable 1:1 inclusion complexes with DCN, with HPβCD showing higher complexation efficiency. Various preparative techniques—ranging from physical mixing to evaporation, kneading, and precipitation—have been employed. Characterization using Fourier transform infrared spectroscopy (FTIR), Differential scanning calorimetry (DSC), and Powder X-ray Diffraction (PXRD) confirmed the predominantly amorphous state of DCN within these complexes, thereby facilitating enhanced dissolution profiles [4,90,109]. Recent innovations include the development of cyclodextrin metal–organic frameworks (CD-MOFs), which provide eco-friendly and biocompatible platforms for drug delivery, owning to their high specific surface area, porosity, and facile fabrication methods [110].

#### 4.2.2. Co-Crystal and Eutectic Formation for Enhanced Bioavailability

Co-crystallization and eutectic formation modify drug crystallinity, resulting in faster dissolution and improved pharmacokinetics. DCN was co-crystallized with 2,4-dihydroxy benzoic acid (DHA) and fumaric acid (FMA) using a mechanochemical grinding method, forming a stable multi-component crystalline phase [106]. PXRD and DSC analyses confirmed reduced crystallinity, improved dissolution in citrate buffer (pH 6.0), and increased GI absorption [106]. Similarly, eutectic formulations of DCN showed enhanced pharmacokinetic properties through altering crystallinity to improve dissolution, as validated through a Reversed-Phase High-Performance Liquid Chromatography bioanalytical method [107]. Another co-crystallization strategy involved β-resorcylic acid via antisolvent crystallization, yielding a 1:3 DCN:β-resorcylic acid cocrystal with superior mechanical properties [74]. This formulation significantly improved compactibility and compressibility, making it suitable for tablet formulations. Additionally, a 3.2-fold, increase in bioavailability was observed, confirming improved systemic absorption [74]. These findings highlight that co-crystals and eutectic formulations not only improve DCN physicochemical properties but also enhance mechanical characteristics, making them ideal for solid oral dosage forms.

#### 4.2.3. Ionic Liquids for Stability and Ocular Drug Delivery

Ionic liquids (ILs) stabilize drug molecules, increase permeability, and improve retention in biological membranes. Using betaine-based (Bet6) and L-carnitine-based (Carn6) ILs, Grassiri et al. formulated DCN into stable nanoaggregates, significantly enhancing its solubility and systemic availability. These IL formulations significantly reduced DCN hydrolysis, extending its half-life and therapeutic action [62]. Additionally, their mucoadhesive properties enhanced ocular retention by preventing rapid drug elimination. Moreover, these ILs demonstrated antibacterial activity against *Staphylococcus aureus* and *Pseudomonas aeruginosa*, broadening their potential use in ocular anti-inflammatory therapies [62]. These combined advantages make IL formulations a promising approach for both oral and topical drug delivery.

As shown in Table 3, each of these modification strategies exhibited varying degrees of effectiveness for DCN.

### 4.3. Advanced Formulations by Route of Administration

**Figure 5:**
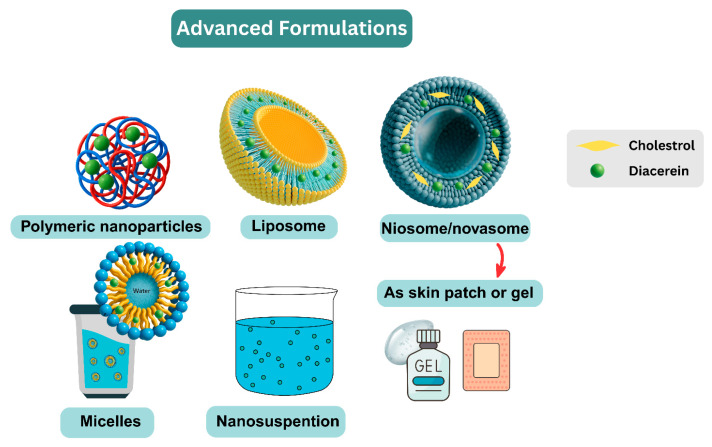
Overview of advanced nanocarrier-based formulations developed for diacerein, including polymeric nanoparticles, liposomes, niosomes/novasomes, micelles, and nanosuspensions, as well as topical gel and patch systems. These platforms aim to improve solubility, stability, targeted delivery, and overall therapeutic performance.

This section is organized by route of administration. Non-oral systems are covered in Section 4.3.1, Section 4.3.2, Section 4.3.3, Section 4.3.4 and Section 4.3.6, whereas oral nanoformulations are consolidated in Section 4.3.5. This avoids overlap with earlier general mechanisms in Section 4.1 and Section 4.2. An overview of advanced nanocarrier-based strategies for diacerein delivery is illustrated in Figure 5.

#### 4.3.1. Vesicular Systems (Topical, Transdermal, Intra-Articular)

Various vesicle-based nanoformulations, including niosomes, bilosomes, novasomes, elastosomes, and hyaluosomes, have been explored to optimize topical, transdermal, and intra-articular delivery of DCN, improving therapeutic outcomes while minimizing systemic side effects. Among recent advances, surfactant-based drug delivery systems have emerged as pivotal tools. Due to their unique amphiphilic structure, comprising both hydrophilic and lipophilic segments, surfactants are highly versatile and have been incorporated into nearly all dosage forms to enhance drug absorption. This capability has spurred extensive research, positioning surfactants as a key player in addressing the challenges of low aqueous solubility [6,111,112].

The adsorption of surfactant molecules from the liquid phase onto solid surfaces is mainly driven by electrostatic and hydrophobic interactions (physical adsorption); However, in some instances, chemical bonds may also form. When this adsorption results in the formation of ordered structures—ranging from monolayers to complex multilayer assemblies—it is often called surface self-assembly. Similarly, in bulk liquids, self-assembly gives rise to the formation of micelles, vesicles, and other higher-order structures [113]. For instance, Pluronic^®^ F127 has been evaluated as a solubilizing agent in SDs of DCN, which demonstrated its potential as an effective carrier for improving the drug’s dissolution profile [6].

To address the delivery challenges posed by DCN, surfactant-based formulations have been extensively investigated as effective vesicular drug delivery systems for enhancing solubility, permeability, and controlled drug release [6]. These surfactant-based vesicles provide protection from enzymatic degradation, improved systemic absorption, and reduced GI side effects. Multifarious topical, transdermal, and oral formulations have been developed, each tailored to optimize the drug’s pharmacokinetic profile and therapeutic potential [15,60,114].

##### Niosomal Formulations

Topical niosomal formulations have demonstrated promising results in treating psoriasis and OA by enhancing drug retention within target tissues, reducing systemic absorption, and prolonging anti-inflammatory activity [115,116]. Moghddam et al. developed a Span 60-based niosomal gel containing DCN for the management of psoriasis. This formulation achieved 83.02% EE with vesicle sizes of 477.8 nm, ensuring deep skin penetration. Franz diffusion studies confirmed a flux of 2.820 μg/cm^2^/h, and Confocal Laser Scanning Microscopy demonstrated drug localization within epidermal layers (30–90 µm depth), supporting sustained therapeutic effects [1]. Similarly, a niosomal gel of DCN for OA treatment, prepared via thin-film hydration (THF) and sonication, achieved 9.52–58.43% EE and controlled drug release over 8 h, optimizing localized retention. Box–Behnken experimental design confirmed surfactant concentration and charge-inducing agents as critical factors influencing vesicle dispersion and drug release [60].

Aziz and co-workers optimized a transdermal niosomal formulation of DCN, prepared via THF, using a three-level central composite design for RA treatment. The optimized formulation achieved 95.63% EE of DCN and vesicle sizes of 436.65 nm, ensuring enhanced penetration and stable vesicular dispersion. Ex vivo permeation studies demonstrated significantly improved drug retention within the skin layers, effectively reducing systemic exposure while maintaining prolonged therapeutic action [117].

For oral administration, niosomal encapsulation strategies protect DCN from enzymatic hydrolysis, improve dissolution rates, and facilitate sustained drug release, thereby enhancing systemic bioavailability. A THF-based niosomal formulation of DCN, incorporating Span 20, Span 60, and cholesterol, achieved an EE of 74.09%, with vesicle sizes 0.5–2.6 µm. The optimized formulation exhibited 93.24% drug release within 60 min, significantly improving absorption for the management of OA [50]. In addition, reverse-phase evaporation niosomes containing DCN extended the T_50_% (time to release 50% of the DCN) up to 10 h, following zero-order kinetic release (R^2^ = 0.9834), providing prolonged systemic exposure and reduced dosing frequency [118].

To accelerate dissolution rates, ultrasonic processing of niosomes was introduced as an eco-friendly alternative, producing smaller vesicles (154–405 nm) with an EE of 82.6% EE for DCN, thereby enhancing drug absorption [42]. Additionally, stability-enhanced niosomal formulations confirmed structural integrity and resistance to enzymatic degradation, supporting prolonged systemic retention [53]. Furthermore, Khan et al. studied a THF-based niosomal formulation of DCN using sorbitan monolaurate and poloxamer 184, which achieved an EE of 90.5%, with particle sizes ranging from 350–1000 nm, ensuring gradual drug release and reduced GI irritation [41].

##### Other Types of Vesicular Systems

Bilosomes, modified niosomal vesicles, incorporate bile salts as edge activators to improve vesicle deformability and penetration [119]. Aziz et al. investigated a THF-based formulation containing DCN for topical application, followed by sonication, which resulted in 100% EE with a vesicle size of 301.65 nm, facilitating superior absorption and retention. ex vivo permeation studies also confirmed enhanced drug transport through the skin, offering a promising alternative to oral DCN, which is limited by first-pass metabolism and hepatic degradation, particularly in older patients [54].

Novasomes, nano-vesicles consisting of free fatty acid, offer improved transdermal drug bioavailability [120]. A novasome formulation containing DCN was formulated using Span 60, cholesterol, and stearic acid. The optimized formulation achieved an EE of 69.415% with a vesicle size of 275.2 nm, ensuring deep tissue penetration for transdermal delivery in the management of OA [114]. Additional studies optimized the cholesterol-to-surfactant ratio, identifying 1:4 as the most efficient, leading to higher transdermal flux and reduced systemic toxicity [57].

Elastosomes, highly deformable non-ionic surfactant-based vesicles, incorporate edge activators such as Brij and Cremophor to enhance drug penetration and systemic retention [121]. Aziz and coworkers. developed a THF-based formulation with an EE 96.25%, representing the optimal DCN loading compared to the oral formulation for managing OA. The formulation exhibited a vesicle size of 506.35 nm and a deformability index of 12.74 g(DI), indicating high flexibility and superior absorption, and achieved a 19-fold increase in transdermal flux compared to conventional suspensions. Histopathological analysis confirmed its non-irritant nature, ensuring safe application for OA treatment [76].

Hyaluosomes, which are hyaluronic acid-based liposomes, can enhance local retention and control release [122]. These were developed by Eladawy et al. for a dual-function platform of intra-articular drug delivery, specifically for OA treatment [8]. The optimized formulation exhibited a particle size of 310 nm, 90.7% EE of DCN, and a zeta potential of −12.2 mV, ensuring colloidal stability and extended joint retention for OA. This delivery system of DCN provided sustained drug release over 48 h (48% retention vs. rapid systemic clearance in oral formulations), reducing joint inflammation and cartilage degradation for in vivo OA models. The significant decrease in TNF-α and IL-1β levels in this system confirmed its strong anti-inflammatory effects, supporting its therapeutic application in OA management [8]. A summary of the formulation parameters and therapeutic advantages of these vesicular nanoformulations is presented in Table 4.

As shown in Table 4, nano-vesicular carriers offer significant potential for improving DCN bioavailability, targeted delivery, and sustained therapeutic effects across various administration routes.

#### 4.3.2. Emulsion and Gel-Based Systems (Topical/Transdermal)

Nanoemulgels, microemulgels, and nanogels serve as stable and efficient drug delivery platforms, optimizing topical and transdermal administration for the treatment of inflammatory disease [122,123,124]. Kazi et al. explored the potential of nanoemulgels, which integrate nanoemulsions into a hydrogel matrix to ensure deep skin penetration and sustained release of DCN. This nanoemulsion-based system, formulated with Carbopol 940, Triacetin: Capmul MCM C8 (2:1 *w*/*w*), and Kolliphor EL:Transcutol-P (1:1 *w*/*w*), achieved over 90% EE and a mean globule size of 10.7 nm, promoting rapid skin penetration and enhanced solubility [25]. Another investigation confirmed that controlled release formulations of DCN led to improved absorption, reduced systemic fluctuations, and decreased dosing frequency. This alternative nanoemulgel delivery system, incorporating oleic acid (8–12%), Tween 80 (10–15%), and propylene glycol (15–25%), demonstrated 93.61% DCN release over 24 h, with a flux of 0.574 µg/cm^2^/h, ensuring extended therapeutic action [21].

Microemulgels were formulated by Pawbake et al. using Capmul MCM C8 (oil), Labrasol (surfactant), and ethanol (cosurfactant), exhibiting high thermodynamic stability and enhanced DCN permeation. A central composite design was employed to optimize the superior formulation, achieving 95% drug release within 8 h, confirming efficient solubilization and diffusion [125].

Nanogels containing DCN, formulated using Carbopol 940 and Eudragit RSPO, were developed via the emulsion solvent diffusion technique, which revealed improved stability and penetration for the topical treatment of arthritis. Following Higuchi’s diffusion-controlled model, the optimized formulation exhibited a particle size of 190.3 nm, an EE OF 83.51%, and a DCN release of 90.13% over 24 h. Ex vivo permeation and in vivo studies in the Freund’s Adjuvant Arthritis model further confirmed significant anti-arthritic efficacy, indicating that these nanogels represent a promising transdermal alternative to conventional oral formulations [56]. A summary of the formulation characteristics and therapeutic benefits of these nano-based gels is presented in Table 5.

These emulsion- and gel-based systems offer diverse advantages, positioning them as promising alternatives for topical and transdermal DCN therapy.

#### 4.3.3. Lipid-Based Nanoformulations (Topical & Intra-Articular)

This subsection covers lipid carriers used primarily for topical and intra-articular delivery (e.g., lipid carriers (NLCs), solid lipid nanoparticles (SLNs), liposomes) to enhance local retention and reduce systemic side effects. Comparative details remain in Table 6. Pawbake and co-workers developed NLC-based gels for topical OA treatment and demonstrated sustained release of DCN over 24 h. They reported enhanced localized retention and anti-inflammatory effects of these gels [23]. Another study, SLNs were formulated using cetyl alcohol (2%) and Tween 80 (0.9%) for oral encapsulation of DCN, which exhibited long-term stability (6 months) and reduced GI side effects, particularly diarrhea, the main clinical limitation of using DCN [126]. Chondroitin sulfate (ChS)-modified SLNs were also designed for intra-articular injections by Jain et al. These formulated SLN could increase cartilage targeting, as long as they sustain drug release for 16 h, in addition to improving rhein retention at the joint site (7.8 ± 1.23 μg/mL vs. 2.9 ± 0.45 μg/mL for free drug) [38].

Liposomes and proliposomes were widely studied for both oral and transdermal administration [127,128,129,130]. Shah et al. developed a liposomal gel formulation that enhanced the skin penetration of DCN by 3.5-fold and improved systemic absorption [24]. In addition, proliposome-based controlled-release tablets composing of Eudragit RS100 and ethyl cellulose achieved 91.13% DCN entrapment and sustained drug release (46.7% over 12 h), suggesting superior pharmacokinetics compared to conventional DCN capsules [70].

A comparative evaluation of various lipid-based nanoformulations of DCN is presented in Table 6. As discussed, these lipid-based formulations represent promising delivery strategies for DCN.

#### 4.3.4. Injectable and Targeted Delivery Systems

Injectable and targeted drug delivery systems enhance localized retention, controlled release, and therapeutic efficacy for intra-articular OA treatment. These systems improve cartilage targeting while reducing systemic side effects and the need for frequent dosing [131,132,133].

Poly lactic-co-glycolic acid (PLGA) microparticles were developed using emulsion-solvent evaporation, which encapsulated rhein with an EE of 63.8%. This formulation ensured biphasic drug release (45% within 24 h, followed by sustained release over 30 days). In vitro anti-inflammatory analysis confirmed significant reductions in IL-1β and reactive oxygen species levels, highlighting the therapeutic potential of PLGA microparticles [52].

Ali et al. developed surface-modified iron oxide microparticles containing DCN, coated with chitosan and hydrolyzed collagen. This delivery system facilitated localized joint retention with high EE value (85.25%) and a sustained release profile, minimizing systemic toxicity. The magnetic targeting capabilities of the formulation resulted in precise drug localization, along with reduced TNF-α and IL-1β levels and enhanced cartilage regeneration [59].

Chondroitin sulfate-modified SLNs (ChS-DC-SLNs) were also designed by Jain et al. for intra-articular injections. This formulation enhanced cartilage targeting and provided sustained drug release for up to 16 h. ChS-DC-SLNs achieved a high EE (93.8%) and significantly improved intra-articular drug retention. Compared to uncoated SLNs (4.62 μg/mL) and free drug (2.9 μg/mL), ChS-modified SLNs demonstrated rhein retention of 7.8 μg/mL at the joint site, resulting in 2.8-fold higher bioavailability and superior cartilage protection [38].

A comparative evaluation of various intra-articular drug delivery systems for DCN is presented in Table 7.

#### 4.3.5. Oral Nano-Formulations of DCN

As detailed earlier (Section 4.1, Section 4.2 and Section 4.3), multiple nano-enabled oral systems—nanosuspensions, SNEDDS, and polymeric/pH-responsive carriers—have been investigated to overcome DCN’s dissolution limits and enzymatic degradation [134]. To avoid redundancy with prior mechanistic descriptions, this subsection focuses on DCN-specific oral outcomes, with a concise comparison in Table 8.

##### Nanosuspensions

Nanosuspensions utilize reduced particle size to increase poorly soluble drugs’ surface area and dissolution rate of poorly soluble drugs [135]. Elsayed et al. developed a hybrid bottom-up/top-down approach, combining high-pressure homogenization and precipitation techniques, to prepare DCN nanosuspensions. Optimized formulations incorporated sodium deoxycholate and sodium dodecyl sulfate, resulting in a significant improvement in DCN dissolution. The best-performing formulation exhibited a 2.23-fold increase in solubility and achieved complete drug dissolution during 1.26 min, compared to 13.65 min for coarse DCN. Pharmacokinetic evaluations also confirmed a 131.4% increase in bioavailability, with a higher peak plasma concentration (C_max_ = 3.8 µg/mL compared to 2.0 µg/mL for commercial formulations of DCN) and faster absorption (T_max_ = 1.2 h vs. 1.7 h). These findings support the potential of nanosuspension formulations to delivery of DCN [51].

Additionally, a coated nanosuspension was investigated to improve mucoadhesion and drug stability. The formulation was prepared using sonoprecipitation followed by lyophilization, with PVP serving as a stabilizer. The chitosan coating enhanced permeability and sustained drug release, achieving an EE of 95.63% and a particle size of 145.4 nm. Ex vivo studies showed increased mucoadhesion and prolonged absorption, leading to a 172.1% higher relative bioavailability than conventional DCN suspensions [49].

##### Self-Nanoemulsifying Drug Delivery Systems

Self-emulsifying drug delivery systems (SEDDS) have emerged as a promising alternative for lipophilic drugs. These isotropic formulations form oil-in-water emulsions upon gentle agitation in the GI tract, offering significant potential for improved drug delivery. The formation of such emulsions facilitates the drug in a solubilized form, and the resulting small droplet size yields a large interfacial area that favors drug absorption [136]. SEDDS formulations typically consist of a blend of lipids (natural or synthetic), nonionic surfactants having high hydrophilic–lipophilic balance values (e.g., Tween 80, Ethoxylated polyglycolide glyceride), and hydrophilic cosolvents/emulsifiers. Although the number of surfactants approved for oral use is relatively limited, nonionic surfactants are preferred due to their lower toxicity and rapid self-emulsifying efficiency [137,138].

DCN was incorporated into a self-nanoemulsifying system, ternary phase diagrams were constructed using oils such as Capryol™90, Miglyol^®^812, and isopropyl myristate, combined with surfactants (Tween^®^80 and Tween^®^20) and cosurfactants (PEG 200 and PEG 300) [93].

A SNEDDS formulation for rhein was optimized using medium-chain triglycerides (Capmul MCM C8) as the oil phase, with Labrasol and Transcutol-P as surfactants and co-surfactants. The optimized SNEDDS exhibited a droplet size of 42.3 nm, leading to complete drug release within 30 min and enhanced systemic absorption. These formulations effectively prevented drug precipitation, improved GI solubility, and minimized dose variability [48].

##### pH-Responsive Hydrogels and Polymeric Microspheres

Biopolymers, derived from natural sources or biological systems, offer a versatile and sustainable option for pharmaceutical formulations owing to their biodegradability, availability, and tunable physicochemical properties [139]. Their unique features have garnered interest in composite materials, where natural fibers frequently serve as reinforcement within biodegradable matrices, yielding entirely environmentally friendly composites [140].

Several studies have investigated the use of biopolymers to improve therapeutic performance in DCN delivery. For instance, GR drug delivery systems have been formulated using swellable polymers such as HPMC K100M and PEO, combined with chitosan and carbopol, to achieve extended therapeutic action [141].

Haseeb et al. explored stimuli-responsive hydrogels and polymeric microspheres for controlled DCN release. These hydrogels minimized gastric degradation and improved sustained absorption in the intestine [61]. Linseed hydrogel-based tablets were fabricated using rhamnogalacturonan polysaccharides to respond to pH-dependent conditions. This formulation prevented premature DCN release in gastric conditions (pH 1.2) and enhanced drug release in intestinal pH (6.8–7.4), thereby ensuring sustained absorption of DCN. The porous nature of the hydrogel structure increased DCN’s hydration and dispersion. It also protected DCN from early conversion to rhein, thereby reducing GI irritation and improving therapeutic efficacy [61].

In another approach, acrylic acid-based pH-responsive polymeric hydrogels containing DCN were formulated to enhance swelling and control drug diffusion. Optimized formulations demonstrated improvement in DCN release in an in vitro study (at pH 7.4), effectively preventing dose dumping and prolonging therapeutic effects [142]. Microsphere formulations using sodium alginate and chitosan as polymeric carriers were also developed via ionotropic gelation, resulting in slow DCN diffusion and continuous DCN retention. The optimized formulation (sodium alginate: chitosan = 1:3) exhibited the lowest release rate with stable drug entrapment [143]. Additionally, DCN-loaded microspheres crosslinked with calcium chloride showed fewer adverse effects [143]. Roy et al. fabricated microspheres containing DCN to evaluate their efficacy in an induced arthritis model in Sprague Dawley rats that showed improved joint function, reduced inflammation, decreased peak plasma concentrations, and reduced GI side effects associated with DCN use [144]. Furthermore, DCN-loaded microspheres prepared by ionotropic gelation using polymers like sodium alginate and chitosan, crosslinked with calcium chloride, led to fewer adverse effects [143].

Table 8 briefly shows the key pharmacokinetic properties and solubility enhancements achieved by nanosuspensions, SNEDDS, and polymeric delivery systems, highlighting the effectiveness of various nanoformulation techniques.

**Table 8:** Comparative analysis of oral nanoformulations for DCN.

Technique	Target Compound	Disease Treated	Solubility Enhancement	Bioavailability Increase	Dissolution/Release Profile	Ref.
Nanosuspension (F12-SDS)	DCN	Osteoarthritis	2.23-fold increase	131.4% increase vs. commercial DCN	1.26 min (complete dissolution)	[51]
Chitosan-Coated Nanosuspensions	DCN	Osteoarthritis	Increased mucoadhesion	172.1% increase vs. conventional DCN	Sustained release	[49]
SNEDDS	Rhein (DCN Metabolite)	Osteoarthritis	Yes	Enhanced systemic absorption	Complete release in 30 min	[48]
Linseed Polysaccharide Hydrogel	DCN	Osteoarthritis	pH-responsive swelling	Sustained drug absorption	Controlled release in intestinal pH	[61]
pH-Responsive Polymeric Hydrogels	DCN	Osteoarthritis	Yes	Controlled swelling	Extended release in pH 7.4	[142]
Ionotropic Gelation Microspheres	DCN	Osteoarthritis	Yes	Controlled drug entrapment	Slow drug diffusion	[143]
Microsphere-Based SR Formulation	DCN	Arthritis	Not Reported	Reduced peak plasma fluctuations	Improved joint function	[144]

Abbreviations: DCN: Diacerein, min: Minute, pH: Potential of Hydrogen, SNEDDS: Self-Nanoemulsifying Drug Delivery System, SDS: Sodium Dodecyl Sulfate, SR: Sustained Release.

#### 4.3.6. Dissolving Microneedles for Percutaneous Drug Delivery

Recently, dissolving microneedles have gained significant attention as a minimally invasive approach for enhancing transdermal drug delivery. These systems create microchannels in the skin, enabling rapid absorption of active agents with reduced systemic side effects [145,146]. Shabbir and coworkers investigated a DCN micro-suspension that was incorporated into HPMC and PVP microneedle matrices for effective percutaneous delivery. The microneedles dissolved within 5 min, releasing 74.39% of the DCN within 24 h. Thermal analysis studies confirmed that the amorphous state of DCN led to improved dissolution. In vivo results using a carrageenan-induced paw edema model also demonstrated significant anti-inflammatory effects and highlighted the therapeutic potential of these dissolving microneedles [58].

Another study investigated microneedle-assisted delivery of an SD of DCN. DCN was pre-treated with Polysorbate 80 or formulated as a PEG 4000-based dispersion before being incorporated into lyophilized HPMC/PVP microneedles to enhance solubility. In vitro results showed that microneedles dissolved rapidly and formed aqueous microchannels, resulting in a 2.43-fold increase in transdermal DCN delivery. It was also reported that over 98% of the drug was absorbed within 24 h. These results indicate that this delivery system significantly improved bioavailability and ensured sustained drug action [147]. Table 9 summarizes the details of microneedle-based delivery systems of DCN.

### 4.4. Enhancing DCN’s Therapeutic Effects Through Synergistic Strategies

Optimizing DCN’s efficacy through synergistic drug combinations has been shown to be a promising way to improve bioavailability, reduce systemic toxicity, and target multiple disease pathways. These combination strategies can include co-administered compounds, lipid-based carriers, nanoemulsion-based formulations, and hydrogels. The outcomes of such synergistic strategies have shown potential benefits in treating neurodegenerative diseases, psoriasis, RA, and OA. Sharma et al. investigated a neuroprotective combined formulation of *Adenium obesum* extract with DCN for Parkinson’s disease and depression. The extract, known for its antioxidant and anti-inflammatory properties, enhanced the effects of DCN, leading to reduced neuronal degeneration, improved hippocampal structure, and improved motor coordination. Antioxidant assays also showed increased resistance to oxidative stress, further supporting its potential role for neurodegeneration therapy applications [2].

A transferosomal hydrogel loaded with DCN and berberine HCl was developed for topical application. This hydrogel enhanced drug retention, penetration, and anti-inflammatory effects. Also, the formulation showed sustained release (88.44% DCN, 81.56% berberine HCl over 24 h) and significantly reduced epidermal thickness and inflammatory cytokines (TNF-α, IL-17A) [148]. A dual-drug transferosomal system using sodium deoxycholate as an edge activator further improved skin penetration and stability. The optimized formulation (a particle size of 110.9 nm, and 91.23% DCN EE) showed enhanced dermal absorption and maintained stability for three months [149].

El-Refaie et al. designed an SLN containing rhein and methotrexate to improve joint targeting and reduce systemic toxicity in the treatment of RA. This nanoformulation (a particle size of ~192 nm, 93% drug EE) showed controlled release (~48% over 24 h) and significantly reduced TNF-α and IL-1β levels. The results suggest this combined strategy offers superior anti-inflammatory effects compared to free drugs [150]. Another study on a binary SLN system (stearic acid, lauric acid, oleic acid) revealed sustained release over 72 h, with temperature-sensitive acceleration at 40 °C for joint-targeted therapy. Gold nanoparticles were incorporated into this SLN system to further reduce inflammation, showing effective cartilage protection in in vivo models [28].

Chattopadhyay and coworkers developed a nanoemulsion-based hydrogel (composed of soybean oil, Tween 80, and poloxamer 407) for transdermal glucosamine-DCN delivery. In vitro results showed stable nanoemulsion formation (81.95 nm, zeta potential: 39.33 mV). In vivo results also demonstrated reduced TNF-α, C-reactive protein, and inflammatory markers, confirming strong chondroprotective effects of this formulation [55].

A clinical trial comparing a combination of chondroitin sulfate and glucosamine sulfate with DCN monotherapy in knee OA found a 71.01% pain reduction in the combination group versus 48.09% with DCN alone. This synergic system showed greater symptomatic relief and joint protection [63].

To summarize the advantages of different combination strategies, Table 10 presents the key pharmacokinetic improvements and therapeutic benefits discussed in this section.

However, future research can be designed on hybrid nanocarrier systems combined with other delivery methods. The integration of nanotechnology with polymeric and stimuli-responsive systems presents a promising strategy to optimize DCN’s anti-inflammatory clinical performance.

## 5. Clinical Potential of DCN in Novel Carriers and Formulations

Various formulations of DCN have demonstrated significant clinical potential for treating inflammatory disease. Following is a brief explanation of the most important clinical trial applications of DCN.

Clinical trials have investigated DCN in dermatological applications, particularly for psoriasis and EB. A clinical study (ClinicalTrials.gov Identifier: NCT03472287) evaluating 1% DCN ointment in EB reported significant improvements in blister reduction and skin barrier function [5]. Unlike in other dermatological conditions, EB patients did not experience issues with drug penetration, making sustained drug release a more critical factor than absorption, which this goal can be achieved with more novel drug delivery systems. This goal can be achieved with novel drug delivery systems. To enable continuous and controlled drug delivery, advanced-release formulations, such as lipid-based carriers and nanoemulgel-based systems, have been developed to provide prolonged skin retention while reducing the need for frequent application [25,114,117]. DCN has been widely orally investigated in OA trials, but its clinical efficacy has been limited by poor bioavailability and GI side effects. As mentioned, a clinical study (ClinicalTrials.gov Identifier:NCT01906801) has explored DCN as a monotherapy or in combination with glucosamine and diclofenac, showing positive effects on pain relief and joint function [151].

Osmotic-controlled release tablets, SD techniques, and chitosan-coated nanosuspensions have been investigated to improve DCN’s dissolution rates for OA treatment. These approaches have reported enhanced DCN bioavailability while minimizing GI irritation [3,6,49]. Advanced formulations such as PLGA microparticles, lipid-based, and SLN-based nanoparticles have demonstrated extended DCN release, increased joint retention, and reduced levels of inflammatory cytokines (TNF-α, IL-1β), supporting their potential for long-term OA management [8,28,52].

DCN has also been explored in clinical trials for ocular conditions (ClinicalTrials. gov Identifier:NCT04351100), particularly ocular surface disease and inflammation [152]. However, poor retention on the ocular surface and rapid clearance have limited its effectiveness in ophthalmic applications. Recent formulation advancements, such as IL-based eye drops, have improved DCN’s ocular retention and bioavailability, making it a promising candidate for chronic inflammatory eye diseases [62]. To provide a comprehensive overview of the clinical evaluation of Diacerein across various diseases, all registered clinical trials listed on ClinicalTrials.gov were systematically reviewed. As summarized in Table 11, at least 30 clinical studies have investigated Diacerein in diverse therapeutic domains, including osteoarthritis, metabolic inflammation, epidermolysis bullosa, ocular inflammatory disorders, diabetes mellitus, rheumatoid arthritis, and thrombocytopenia. These trials encompass Phase 1 to Phase 4 investigations and collectively highlight the drug’s broad therapeutic footprint and continued clinical relevance [27]. Integrating these clinical findings with formulation advancements underscores the importance of developing optimized delivery systems to fully harness the therapeutic potential of Diacerein. Future studies should focus on combining these synergic systems with clinical trial outcomes and regulatory approval processes to facilitate the commercialization of these optimized formulations.

## 6. Discussion

### 6.1. Regulatory and Clinical Translation Challenges

Despite its categorization as a SYSADOA, DCN remains unapproved by the U.S. FDA and is subject to restricted use by the European Medicines Agency (EMA) due to its significant adverse effects, particularly dose-dependent diarrhea, hepatotoxicity, and hyperkalemia in elderly patients (EMA, 2014 [153]). These regulatory barriers have motivated research efforts toward advanced formulation strategies that minimize systemic toxicity, improve site-specific delivery, and optimize pharmacokinetics [126].

**Figure 6:**
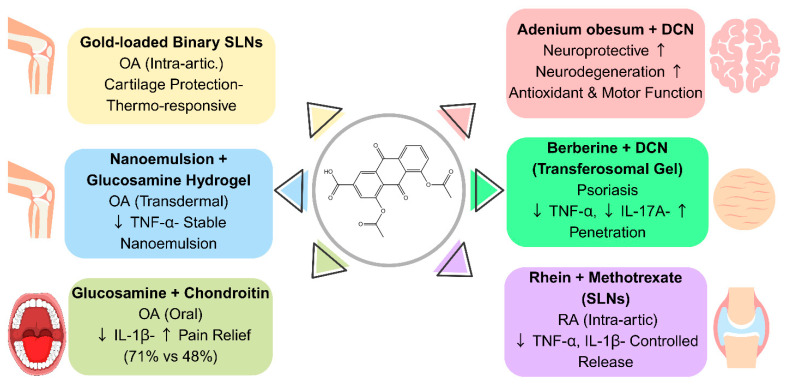
Examples of combinational and hybrid strategies involving diacerein, including gold-loaded solid lipid nanoparticles for osteoarthritis, glucosamine-based nanoemulsions and hydrogels, berberine-loaded transferosomal gels for psoriasis, rhein–methotrexate SLNs for rheumatoid arthritis, and glucosamine–chondroitin oral formulations for enhanced pain relief.

Among these strategies, solid lipid nanoparticles (SLNs) have demonstrated notable promise, with QbD-optimized oral formulations enhancing anti-inflammatory efficacy while reducing hepatic enzyme levels in preclinical models [126]. Likewise, niosomal gels for topical application have been designed to reduce gastrointestinal exposure while maintaining therapeutic efficacy at the site of action, showing favorable ex vivo permeation and in vivo anti-inflammatory outcomes [1,2,114,117,148,149]. More innovative approaches, such as self-dissolving microneedle-assisted percutaneous systems, have further enhanced therapeutic outcomes, as reported by Shabbir et al., who noted reduced paw edema and fewer diarrheal episodes compared to conventional oral formulations [147]. In addition, several combinational and hybrid strategies, where DCN is co-formulated with other bioactives or nanocarriers, have been proposed to achieve synergistic therapeutic effects across osteoarthritis, rheumatoid arthritis, psoriasis, and neurodegenerative conditions. Representative examples are illustrated in Figure 6. Such hybrid approaches highlight future opportunities for enhancing both local efficacy and systemic tolerability of DCN therapies.

Despite these advances, translation into clinical and industrial practice remains restricted. Most reported nanoformulations suffer from formulation complexity, scale-up challenges, and a lack of ICH-compliant stability studies (Q1A(R2)). In addition, standardized and validated bioanalytical methods for quantifying rhein in biological samples are scarce, limiting reproducibility across laboratories. Regulatory considerations also add layers of difficulty: many nanocarriers (e.g., microneedle systems, lipid–polymer hybrids) fall under drug–device combination categories, requiring dual regulatory evaluation and expanded toxicological profiling according to FDA nanotechnology guidance [154].

Another key limitation is the absence of late-phase clinical evidence. While numerous in vitro and in vivo studies have highlighted improved therapeutic efficacy, no diacerein nanoformulation has advanced beyond early-phase trials. This highlights the urgent need for harmonized regulatory frameworks, robust pharmacokinetic and toxicological modeling, and validated analytical pipelines. In particular, physiologically based pharmacokinetic (PBPK) models and advanced simulation tools could bridge the gap between preclinical efficacy and clinical translation by predicting dissolution, absorption, and systemic exposure across patient populations.

In this context, artificial intelligence (AI) and machine learning (ML) are increasingly recognized as transformative tools in the rational design and development of nanocarrier-based diacerein formulations. AI-driven Quality by Design (QbD) frameworks facilitate prediction and optimization of key formulation attributes—such as particle size, encapsulation efficiency, and release kinetics—through data-informed modeling [155,156]. ML models like artificial neural networks (ANNs) and support vector machines (SVMs) have shown promise in enhancing formulation robustness and minimizing experimental iterations, especially for poorly water-soluble anti-inflammatory drugs [157,158]. More recently, AI-assisted physiologically based pharmacokinetic (PBPK) modeling has enabled the prediction of systemic exposure and biodistribution of nanoformulations, potentially bridging the gap between preclinical and clinical translation [159,160]. Moreover, ML-driven platforms such as Excipient Prediction Software (ExPreSo; a machine-learning framework trained on formulation data from 335 approved biopharmaceutical products for excipient prediction) offer opportunities for virtual screening of safe and efficient carriers tailored to the physicochemical properties of diacerein [161]. These technologies are anticipated to accelerate development timelines, reduce formulation variability, and support scalable, patient-tailored delivery systems.

To synthesize the comparative performance of DCN formulation strategies, physicochemical approaches (e.g., amorphization, co-crystals, and cyclodextrin complexation) primarily improve dissolution kinetics and hydrolytic stability. In contrast, nanocarrier-based platforms further enhance these improvements by facilitating enhanced membrane transport, prolonging intra-tissue retention, and enabling site-specific targeting. Integrating evidence from multiple studies suggests that no single formulation fully addresses all pharmacokinetic limitations of DCN; instead, a combined optimization of solubility, stability, permeability, and controlled release is required to achieve consistent therapeutic performance.

Given these mechanistic insights, emerging research should focus on hybrid or multifunctional systems that simultaneously address solubility, metabolic instability, and tissue-specific delivery. For example, combining amorphous solid dispersions with mucoadhesive nanoparticles, or co-crystal engineering with lipid-based nanocarriers, may provide more comprehensive control over both dissolution and systemic exposure. Additionally, the incorporation of predictive computational tools, including PBPK-guided formulation optimization, may significantly reduce translational uncertainty and streamline progression to clinical evaluation.

In summary, nanotechnology holds considerable potential to overcome the long-standing pharmacokinetic and safety limitations of DCN. However, successful clinical and industrial deployment will depend on addressing several unmet needs: (i) establishing validated bioanalytical protocols for rhein and DCN metabolites, (ii) developing scalable and reproducible manufacturing workflows, (iii) implementing ICH-compliant stability programs, and (iv) integrating computational and translational modeling with early-phase clinical design. These strategic developments, alongside robust clinical trials, will be essential to advance diacerein nanoformulations from experimental innovation to practical therapeutic reality.

### 6.2. Comparative Performance of Diacerein Formulation Strategies

The comparative overview in Table 12 demonstrates that physical modification strategies—such as solid dispersions, nanosuspensions, and certain amorphization-based approaches—primarily enhance dissolution rate and wettability, leading to moderate improvements in bioavailability. These enhancements result mainly from reduced crystallinity, improved particle–solvent interactions, and increased surface area. In contrast, true physicochemical strategies—including co-crystals, cyclodextrin inclusion complexes, and eutectic systems—provide more robust improvements in solubility and hydrolytic stability by altering the molecular environment of diacerein through intermolecular interactions and host–guest complexation. Nanocarrier-based systems (niosomes, bilosomes, elastosomes, SLNs, NLCs, hyaluosomes, microneedle-assisted platforms) extend these benefits by enabling superior membrane permeation, enhanced tissue retention, reduced gastrointestinal exposure, and—in several studies—attenuation of diarrhea and systemic toxicity. These carriers consistently demonstrated improved anti-inflammatory, chondroprotective, and pharmacokinetic outcomes in vivo. Hybrid delivery systems, such as dual-drug nanoparticles or co-delivery transferosomes, exhibited synergistic therapeutic effects through simultaneous modulation of complementary inflammatory pathways and improved targeting efficiency. Collectively, these data confirm that no single formulation platform resolves all biopharmaceutical limitations of diacerein. Instead, the most promising approach appears to be combining solubility enhancement with controlled release and tissue targeting to achieve consistent therapeutic performance and translational potential.

## 7. Future Directions and Conclusions

Research on DCN is progressing rapidly, offering numerous opportunities to enhance its pharmacokinetic profile, therapeutic efficacy, and patient adherence through advanced delivery strategies. In this review, both physicochemical modification strategies and nano-enabled delivery platforms were comparatively evaluated to clarify how each contributes to solubility enhancement, hydrolytic stability, permeability, and controlled release. Looking forward, several priorities can be identified. First, lipid-based carriers and hybrid vesicular systems should be refined for improved stability, controlled release, and disease-specific targeting. Compared with purely physicochemical approaches, these advanced carriers show superior benefits in enhancing membrane permeation and reducing colonic hydrolysis, making them among the most promising candidates for translational development. Combining these nanocarriers with penetration enhancers or bioactive peptides may enhance local action while reducing systemic toxicity. Second, oral formulations require further optimization to address the persistent challenges of low bioavailability and gastrointestinal irritation. Future work should focus on developing controlled-release platforms, optimizing polymer selection for solid dispersions, and validating computational models such as PBPK to guide dose adjustments across diverse patient populations. Third, alternative delivery routes—including ocular, intra-articular, and transdermal systems—hold significant potential for improving local drug retention and therapeutic outcomes. Rigorous preclinical validation and long-term clinical studies will be critical to confirm their safety and efficacy. Finally, computational and molecular modeling approaches should be integrated with experimental research to streamline formulation design, predict drug–drug interactions, and guide regulatory readiness. These tools can also help bridge mechanistic insights with clinical applicability, addressing a key gap identified across current DCN research.

Overall, DCN remains a valuable anti-inflammatory and disease-modifying drug. By aligning physicochemical optimization with advanced nanotechnology and supporting these developments with robust analytical, regulatory, and clinical frameworks, next-generation DCN therapies can achieve improved efficacy, reduced adverse effects, and broader clinical adoption in the management of inflammatory and degenerative diseases.

## Figures and Tables

**Table 2:** Comparative analysis of physical strategies for DCN solubility/bioavailability enhancement.

Formulation Technique	Disease Treated	Delivery Route	Solubility Enhancement	Bioavailability Improvement	Drug Release Profile	Ref.
Solid Dispersion (PEG, PVP, Pluronic^®^ F127, Mannitol, PVP K30)	Osteoarthritis	Oral	8.6–10.83-fold increase	2.66–4.04-fold AUC increase	Immediate release	[3,6,20,40,100,101]
Fast-Dissolving Tablets (FDTs)	Osteoarthritis	Oral	Yes	Faster dissolution	Immediate release	[39,101,102]
Immediate-Release (IR) Formulation	Osteoarthritis	Oral	90% release in 1 h	1.7-fold AUC increase	Rapid absorption	[103]
Gastro-Retentive (GR) Formulation	Osteoarthritis	Oral	96% release in 2 h	1.2-fold AUC increase	Prolonged gastric retention	[103]
Matrix-Based SR Tablets	Osteoarthritis	Oral	Controlled	Extended half-life	Sustained release (8–24 h)	[3,104]
Fluid Bed Coated Microspheres	Osteoarthritis	Oral	Yes	Prolonged T_max_	Controlled release	[105]

Abbreviations: AUC: Area under the curve, FDTs: Fast-dissolving tablets, GR: Gastro-retentive, IR: Immediate-release, PEG: Polyethylene glycol, PVP: Polyvinylpyrrolidone, SR: Sustained-release, Tmax: Time to reach maximum plasma concentration.

**Table 3:** Comparative analysis of physicochemical strategies for DCN enhancement.

Strategy	Disease Treated	Delivery Route	Solubility Enhancement	Bioavailability Improvement	Key Advantages	Ref.
Cyclodextrin Complex (HPβCD, Freeze-Drying)	Osteoarthritis	Oral (FDT)	100% dissolution in 5 min	50% hydrolysis reduction	Enhances solubility, improves patient compliance	[7]
Cyclodextrin Inclusion Complex (HP-β-CD)	Osteoarthritis	Oral	4.36 mg/mL solubility	99.95% drug release in 60 min	Enhanced dispersibility, improved stability	[4]
Co-Crystal & Eutectic Formation (DHA, FMA)	Osteoarthritis	Oral	Increased solubility	Faster dissolution in citrate buffer	Reduced GI side effects, improved oral absorption	[106]
Eutectic System	Osteoarthritis	Oral	Yes	Improved absorption	Alters crystallinity, enhances dissolution	[107]
Co-Crystallization with β-Resorcylic Acid	Osteoarthritis	Oral (Cocrystal Tablet)	Yes	3.2-fold AUC increase	Improves tabletability, increases bioavailability	[74]
Ionic Liquid-Based Formulations (Bet6, Carn6)	Osteoarthritis, Ocular Inflammation	Oral, Topical	Stabilized formulation	Extended ocular retention, slowed hydrolysis	Antimicrobial activity, enhanced permeability	[62]

Abbreviations: AUC: Area under the curve, Bet6, Carn6: Betaine- and carnitine-based ionic liquids, DHA: 2,4-Dihydroxybenzoic acid, FDT: Fast-dissolving tablet, FMA: Fumaric acid, GI: Gastrointestinal, HPβCD: Hydroxypropyl-beta-cyclodextrin.

**Table 4:** Comparative analysis of vesicular drug delivery systems for DCN.

Formulation Type	Delivery Route	Entrapment Efficiency	Particle Size (nm/µm)	Drug Release Profile	Ref.
Niosomal Gel (Psoriasis)	Topical	83.02%	477.8 nm	Higuchi-model, sustained release	[1]
Niosomal Gel (Osteoarthritis)	Topical	9.52–58.43%	7.33–23.72 µm	Controlled release (8 h)	[60]
Niosomes for Transdermal Therapy	Transdermal	95.63%	436.65 nm	Enhanced penetration & retention	[117]
TFH-Based Niosomes for Oral Therapy	Oral	74.09%	0.5–2.6 µm	93.24% release in 60 min	[50]
Reverse-Phase Evaporation Niosomes	Oral	79.8%	0.608–1.010 µm	Sustained release (T50% = 10 h), zero-order kinetics	[118]
TFH-Based Niosomes (Controlled Release)	Oral	90.5%	350–1000 nm	Gradual drug release prevents rapid metabolism	[41]
Ultrasonic Processing (UP) Niosomes	Oral	82.6%	154–405 nm	Faster dissolution, improved absorption	[42]
Stability-Enhanced Niosomes	Oral/Transdermal	Not reported	Not reported	Prolonged retention, degradation-resistant	[53]
Bilosomes	Transdermal	100.00%	301.65 nm	Enhanced permeability and prolonged retention	[54]
Novasomes (Span 60, Cholesterol, Stearic Acid)	Transdermal	69.415%	275.2 ± 2.68 nm	Improved transdermal absorption and bioavailability	[114]
Novasomes (Optimized Ratio 1:4, Span 60)	Transdermal	High	Not reported	Enhanced permeability, reduced systemic toxicity	[57]
Elastosomes (Edge Activators: Brij, Cremophor)	Transdermal	96.25%	506.35 nm	19-fold higher transdermal flux, sustained release	[76]
Hyaluosomes (HA-Based Liposomes)	Intra-Articular	90.7%	310 nm	Sustained release (48% over 48 h), reduced TNF-α & IL-1β	[8]

Abbreviations: HA: Hyaluronic acid, IL-1β: Interleukin-1 beta, T50%: Time to release 50% of the drug, TFH: Thin-film hydration, TNF-α: Tumor necrosis factor-alpha, UP: Ultrasonic processing.

**Table 5:** Comparative analysis of emulsion and gel-based nanoformulations for DCN.

Formulation Type	Delivery Route	Entrapment Efficiency	Particle Size (nm)	Drug Release Profile	Ref.
Nanoemulgel (Carbopol 940, Kolliphor EL, Transcutol-P)	Transdermal	>90%	10.7 nm	Sustained drug release, improved absorption	[25]
Nanoemulgel (Oleic Acid, Tween 80, Propylene Glycol)	Topical	Not reported	104.3 nm	93.61% release over 24 h, prolonged action	[21]
Microemulgel (Capmul MCM C8, Labrasol, Ethanol)	Topical	Not reported	Not reported	95% drug release in 8 h, stable formulation	[125]
Nanogel (Carbopol 940, Eudragit RSPO)	Topical	83.51%	190.3 nm	90.13% drug release over 24 h	[56]

Abbreviations: Carbopol 940: A cross-linked polymer used as a gelling agent, DCN: Diacerein, Kolliphor EL: Polyoxyl castor oil surfactant, nm: Nanometers, RSPO: Eudragit^®^ RS PO, a water-insoluble but permeable polymer, Transcutol-P: Diethylene glycol monoethyl ether (solubilizer).

**Table 6:** Comparative analysis of lipid-based nanoformulations for DCN.

Formulation Type	Delivery Route	Entrapment Efficiency	Particle Size (nm)	Drug Release Profile	Ref.
NLC-Based Gel	Topical	Not reported	Not reported	Sustained release over 24 h	[23]
SLN-Based Liquid Formulation (Cetyl Alcohol, Tween 80^®^)	Oral	Not reported	Not reported	6-month stability, reduced diarrhea	[126]
Proliposome & Liposomal Gel (Carbopol-934, Lecithin–Cholesterol)	Oral/Transdermal	Not applicable	385.1–762.8 nm	Sustained drug release, improved permeability	[24]
Proliposomes (Controlled-Release Tablets, Eudragit RS100, Ethyl Cellulose)	Oral	91.13%	Not reported	Sustained drug release (46.7% over 12 h)	[70]

Abbreviations: Carbopol-934: A cross-linked polyacrylic acid polymer used as a gelling agent, Ethyl Cellulose: A cellulose derivative used for controlled-release formulations, Eudragit RS100: A cationic copolymer based on dimethylaminoethyl methacrylate, butyl methacrylate, and methyl methacrylate, Lecithin–Cholesterol: A combination of lecithin (a phospholipid) and cholesterol used in liposomal formulations, NLC: Nanostructured Lipid Carrier, nm: Nanometer, SLN: Solid Lipid Nanoparticle, Tween 80^®^: Polyoxyethylene (20) sorbitan monooleate, a nonionic surfactant.

**Table 7:** Comparative analysis of injectable and targeted drug delivery systems for DCN.

Formulation Type	Delivery Route	Entrapment Efficiency	Particle Size (µm/nm)	Drug Release Profile	Ref.
PLGA Microparticles	Intra-Articular	63.8%	4.23 ± 0.87 µm	Biphasic: 45% in 24 h, sustained over 30 days	[52]
Surface-Modified Iron Oxide Microparticles	Intra-Articular	85.25%	1.54 µm	Prolonged drug retention, targeted delivery, reduced TNF-α & IL-1β	[59]
Chondroitin Sulfate-Modified SLNs (ChS-DC-SLN)	Intra-Articular	93.8%	396 ± 2.7 nm	Sustained release up to 16 h, 2.8-fold higher bioavailability	[38]

Abbreviations: ChS-DC-SLN: Chondroitin Sulfate-Modified Diacerein Solid Lipid Nanoparticle, PLGA: Poly lactic-co-glycolic acid, h: Hour, IL-1β: Interleukin-1 beta, nm: Nanometer, µm: Micrometer, SLN: Solid Lipid Nanoparticle, TNF-α: Tumor Necrosis Factor-alpha.

**Table 9:** Comparative analysis of microneedle-based delivery systems for DCN.

Technique	Target Compound	Disease Treated	Delivery Route	Bioavailability Improvement	Release Profile	Key Benefits	Ref.
Dissolving Microneedles	DCN	Inflammatory Disorders	Percutaneous	Enhanced transdermal penetration	74.39% release in 24 h	Non-invasive delivery, improved local absorption	[58]
Microneedle-Assisted Solid Dispersion	DCN	Osteoarthritis	Percutaneous	2.43-fold increase in absorption	98% release in 24 h	Rapid dissolution, enhanced skin permeability	[147]

Abbreviations: DCN: Diacerein, h: Hour.

**Table 10:** Comparative analysis of synergistic formulations and drug delivery systems for DCN.

Technique	Target Condition	Delivery Route	Solubility Enhancement	Bioavailability Improvement	Therapeutic Effect	Ref.
Carrier-Based (A. obesum Co-administration)	Parkinson’s Disease, Depression	Oral	Not Applicable	Improved neuroprotection	Enhanced behavioral and antioxidant responses	[2]
Transferosomal Gel (DCN + Berberine HCl)	Psoriasis	Topical	Not Applicable	Improved spreadability & drug retention	Reduced epidermal thickness, TNF-α & IL-17A suppression	[148]
Dual Transferosomes (DCN + Berberine HCl)	Psoriasis	Topical	Not Applicable	91.23% entrapment efficiency	Enhanced skin penetration, 24-h sustained drug release	[149]
SLNs (Rhein + Methotrexate)	Rheumatoid Arthritis	Oral	Sustained release (~48% over 24 h)	Increased joint targeting	Reduced TNF-α, IL-1β, and inflammation	[150]
Binary SLNs (Thermoresponsive)	Osteoarthritis	Oral & Intra-Articular	Sustained release over 72 h	Enhanced joint retention	Reduced TNF-α & IL-1β	[28]
Nanoemulsion-Loaded Hydrogel	Osteoarthritis	Transdermal	Yes	Stable nanoemulsion formulation	Reduced inflammatory biomarkers	[55]
Chondroitin & Glucosamine Combination	Osteoarthritis	Oral	No	Not applicable	71.01% pain reduction vs. 48.09% with DCN	[63]

Abbreviations: DCN: Diacerein, HCl: Hydrochloride, IL-17A: Interleukin-17A, IL-1β: Interleukin-1 beta, h: Hour, SLN: Solid Lipid Nanoparticle, TNF-α: Tumor Necrosis Factor-alpha.

**Table 11:** Comprehensive list of completed and ongoing ClinicalTrials.gov studies involving Diacerein [27].

Number	NCT Number	Study Title	Conditions	Interventions	Phases
1	NCT01906801	Clinical Efficacy of Glucosamine Plus Diacerein Versus Mono-therapy of Glucosamine	Osteoarthritis|Glucosamine|Diacerein	DRUG: Glucosamine sulfate|DRUG: Diacerein|DRUG: Placebo (for Diacerein)	PHASE4
2	NCT04351100	Efficacy of Diacerein on Ocular Surface Disease in Degenerative Arthritis Patients	Ocular Surface Disease|Osteoarthritis|Diacerein	DRUG: Diacerein	
3	NCT00685542	Effect of Diacerein on Hand Osteoarthritis	Osteoarthritis	DRUG: diacerein|DRUG: placebo	PHASE4
4	NCT03472287	To Evaluate the Pharmacokinetics of Diacerein and Rhein After Maximum Use in Patients With Epidermolysis Bullosa (EB)	Epidermolysis Bullosa (EB)|Epidermolysis Bullosa Simplex|Dystrophic Epidermolysis Bullosa|Junctional Epidermolysis Bullosa	DRUG: Diacerein 1% Ointment	PHASE1
5	NCT01298882	Diacerein on Insulin Secretion in Diabetes	Type 2 Diabetes Mellitus|Overweight|Obesity	DRUG: Diacerein|OTHER: Placebo	PHASE2
6	NCT00445276	Symptomatic Efficacy of Diacerein in Knee Osteoarthritis	Knee Osteoarthritis	DRUG: Diacerein	PHASE4
7	NCT04917679	Eltrombopag Plus Diacerein vs. Eltrombopag in Adult ITP	Thrombocytopenia	DRUG: Eltrombopag plus diacerein|DRUG: Eltrombopag	PHASE2
8	NCT05226754	Study Design of the Diacerein in Patients With COVID-19	COVID-19	DRUG: Diacerein|DRUG: placebo capsules	PHASE2
9	NCT00451360	Chondromodulating Effect of Diacerein in Osteoarthritis of the Hip	Hip Osteoarthritis	DRUG: diacerein	PHASE3
10	NCT00440661	Exploration of the Synovial Fluid Inflammation Mediators Under Diacerhein in Knee Osteoarthritis	Osteoarthritis	DRUG: Diacerhein	PHASE4
11	NCT07199933	Diacerein in the Treatment of Metabolic Dysfunction-Associated Steatotic Liver Disease	MAFLD	DRUG: Diacerein 50 mg Capsule|DRUG: Placebo 50 mg	NA
12	NCT06308068	Bioequivalence Study of Diacerein 50 mg Capsule in Healthy Thai Volunteers	Healthy Volunteer	DRUG: Diacerein 50 mg Capsule	PHASE1
13	NCT02060552	Immune Molecular and Inflammatory Cytokines Dysfunction Analysis in Gout Patients With Different Urate Levels	Primary Gout	DRUG: Diacerein|DRUG: Colchicine|DRUG: Febuxostat	PHASE4
14	NCT04318041	Evaluation of the Structural Modification Effect of Diacerein (ArtrodarÂ^®^) in Knee Osteoarthritic Patients	Knee Osteoarthritis	DRUG: Artrodar|DRUG: Placebos	PHASE3
15	NCT02242149	Efficacy Study of Diacerein on Glycemic Control and Liver Fat in Type 2 Diabetes Subjects	Diabetes Mellitus, Type 2|Non-alcoholic Fatty Liver Disease	DRUG: Diacerein|DRUG: Placebo	PHASE3
16	NCT03472547	A Study of Sensitizing Potential of Diacerein 1% Ointment in Healthy Subjects	Healthy	DRUG: diacerein 1% ointment	PHASE1
17	NCT03472534	A 21 Day Irritation Study in Healthy Volunteers With Diacerein 1% Ointment	Healthy	DRUG: diacerein 1% ointment	PHASE1
18	NCT01120015	Diacerein as Adjuvant to Diclofenac Sodium in Indian Patients of Osteoarthritis (OA) Knee	Osteoarthritis|Osteoarthritis, Knee	DRUG: diacerein	PHASE4
19	NCT02688400	Effect of Diacerein vs. Celecoxib on Symptoms and Structural Changes in Symptomatic Knee Osteoarthritis	Osteoarthritis|Osteoarthritis, Knee	DRUG: Diacerein|DRUG: Celecoxib|DRUG: Placebo	PHASE3
20	NCT02177643	Effect of Diacerein in the Metabolic Control of Patients With DM Type 2 and Secondary Failure to Metformin	Diabetes-Related Complications|Diabetes Mellitus, Type 2|Insulin Resistance	DRUG: Diacerein|DRUG: Placebo	PHASE2
21	NCT03754634	A Prospective, Open, Multicenter Clinical Trial of Eltrombopag Combined With Diacerein in Eltrombopag-Inefficient or Relapsed ITP	Thrombocytopenia	DRUG: Eltrombopag and Diacerein	PHASE2
22	NCT03154333	Safety and Efficacy of Diacerein 1% Ointment for Subjects With Epidermolysis Bullosa Simplex (EBS)	Epidermolysis Bullosa Simplex	DRUG: diacerein 1% ointment|DRUG: A placebo ointment	PHASE2
23	NCT03208309	Effect of Diacerein in the Metabolic Control of Patients With DM Type 2 and Secondary Failure to Metformin	Complications of Diabetes Mellitus|Diabetes Mellitus|Diabetes Mellitus, Type 2	DRUG: Diacerein|DRUG: Placebo	PHASE2
24	NCT03473184	A 4-Day Study to Evaluate the Photoxicity of Diacerein 1% Topical Ointment in Healthy Volunteers	Healthy	DRUG: Diacerein 1% ointment	PHASE1
25	NCT03473197	A 6-Week Study to Evaluate the Photoallergic Potential of Diacerein 1% Ointment in Healthy Volunteers	Healthy	DRUG: Diacerein 1% ointment	PHASE1
26	NCT03404479	Trial to Evaluate Efficacy and Safety of Combination of Diacerein and Celecoxib Administered in Patients With Knee OA	Knee Osteoarthritis	DRUG: Diacerein|DRUG: Celecoxib	PHASE4
27	NCT03389308	Long-term Open-label Study Evaluating Safety of Diacerein 1% Ointment Topical Formulation in Subjects With Epidermolysis Bullosa Simplex	Epidermolysis Bullosa|Epidermolysis Bullosa Simplex	DRUG: diacerein 1% ointment	PHASE2
28	NCT06912035	Efficacy of Diacerein Supplementation on Interleukin-1Î^2^, Hs-CRP, TNF-Î± Levels and Glycemic Control in Uncontrolled Type 2 Diabetes Mellitus Patients at Dr. Mohammad Hoesin General Hospital Palembang	Uncontrolled Diabetes|Diabetes Mellitus Type 2	DRUG: Diacerein 50 mg Capsule|DRUG: Placebo	PHASE2|PHASE3
29	NCT01264211	Safety and Efficacy of the Combination of Diacerein 100 mg Daily and MTX Versus MTX Alone in the Treatment of Early Rheumatoid Arthritis (RA)	Rheumatoid Arthritis	DRUG: Diacerein|DRUG: Placebo	PHASE2
30	NCT06073132	An International, Multicenter, Randomized, Double-Blind, Parallel Group, Vehicle-Controlled, Phase 2/3 Study With Open-Label Extension Evaluating the Efficacy and Safety of Diacerein 1% Ointment for the Treatment of Generalized Epidermolysis Bullosa Simplex (EBS)	Generalized Epidermolysis Bullosa Simplex	DRUG: AC-203|DRUG: Vehicle	PHASE2|PHASE3

Abbreviations: OA: Osteoarthritis, RA: Rheumatoid Arthritis, EBS: Epidermolysis Bullosa Simplex, EB: Epidermolysis Bullosa, DM: Diabetes Mellitus, MAFLD: Metabolic Dysfunction-Associated Steatotic Liver Disease, ITP: Immune Thrombocytopenic Purpura, NCT: National Clinical Trial, Hs-CRP: High-sensitivity C-reactive protein, MTX: Methotrexate, COVID-19: Coronavirus Disease 2019, PHASE1/2/3/4: Phases of clinical trials, NA: Not Applicable.

**Table 12:** Comparative Summary of Diacerein Formulation Strategies and Their Functional Outcomes.

Formulation Type		Delivery Vehicle/System	Physicochemical/Encapsulation Improvement	Therapeutic/PK Outcomes	Mechanistic Correlation	Ref.
Physical strategies	Osmotically controlled oral system (ternary SD + asymmetric osmotic pump)	Ternary SD (Pluronic F127 + Solutol HS15 + PEG 35K) compressed into Opadry^®^ CA–coated asymmetric osmotic pump tablets	Solubility ↑ to 70.2 µg/mL; dissolution 79.28% at 60 min; intrinsic dissolution rate ↑; zero-order 24-h release	Bioavailability ↑ ~2.8-fold in vivo (AUC 28.84 vs. 10.39 ng·h/mL)	Amorphization + surfactant-enhanced wetting ↑ absorption; osmotic pump ensures constant release → improved systemic exposure	[3]
	Solid dispersion–loaded oral tablets	Pluronic^®^ F68 solid dispersion (1:3 drug:carrier) prepared via rotavap and compressed into tablets	Solubility ↑ to 187.6 µg/mL (vs. 22.5 µg/mL); dissolution efficiency ↑ 4.04-fold; dissolution rate ↑ 6.6-fold; 100% release in 2 min; tablet dissolution rate ↑ 12.5-fold vs. marketed	Bioavailability ↑ 2.66-fold vs. marketed product	Amorphization + surfactant-enhanced wetting ↑ solubility and dissolution → rapid absorption; optimized SD matrix prevents crystallinity → higher systemic exposure	[101]
	Solid dispersion system (hydrophilic polymer-based)	Solvent-evaporated SD using hydrophilic polymers optimized via I-Optimal design	Dissolution efficiency ↑ 10.83-fold (15 min) and ↑ 3.42-fold (60 min); MDT ↓ 6-fold; complete amorphization confirmed by DSC/XRD/SEM	Relative bioavailability ↑ 229% (adults) and 262% (geriatrics) vs. plain DCN (PBPK predicted)	Amorphization + polymer-enhanced wettability → faster dissolution → higher simulated plasma exposure; PBPK correlation indicates improved oral bio-performance across populations	[40]
	Orally disintegrating tablets (ODTs) containing optimized solid dispersion	ODTs formulated with co-processed excipients (Prosolv^®^ ODT, Pharmaburst^®^ 500, F-melt^®^) loaded with optimized DCN SD	Dissolution efficiency ↑ 1.50-fold (10 min) and ↑ 1.12-fold (30 min); MDT ↓ 2-fold vs. Diacerein^®^ capsules; rapid wetting/disintegration	Significant ↑ in anti-inflammatory response (higher edema inhibition, *p* < 0.0465); faster onset at 0.5 h vs. Diacerein^®^ capsules	Rapid oral cavity dissolution → reduced DCN reaching colon → less rhein formation; improved solubility + fast disintegration → enhanced absorption & early therapeutic effect	[39]
Physicochemical strategies	Cyclodextrin inclusion complexes (β-CD and HP-β-CD)	Kneaded 1:1 inclusion complexes of DCN with β-CD and HP-β-CD; HP-β-CD used for fast-disintegrating tablets	Increased aqueous solubility; improved dissolution rate in pH 6.8 buffer; enhanced amorphousness confirmed by DSC/XRD/FTIR; stable host–guest complex formation	Improved in vitro release vs. pure drug and comparable to marketed formulation; expected ↓ colonic DCN → ↓ rhein-associated diarrhea	Cyclodextrin cavity entraps DCN → reduced crystallinity + enhanced wettability → faster dissolution → reduced unabsorbed fraction reaching colon → mitigation of GI adverse effects	[4]
	Cyclodextrin inclusion complex (HP-β-CD)	1:1 DCN/HP-β-CD complexes prepared via physical mixture, kneading, co-evaporation, and freeze-drying; evaluated in aqueous and solid state	Improved solubility and dissolution in simulated intestinal fluid; enhanced amorphousness confirmed by DSC/FTIR; binding constants validated via spectroscopic, UV, and HPLC methods; stabilized molecular interaction with CD cavity	No in vivo PK reported; complexation shown to modify hydrolysis kinetics of DCN → potential stabilization against premature degradation	HP-β-CD encapsulation improves drug–carrier binding, reduces crystallinity, and modulates DCN hydrolysis → improved dissolution behavior and more predictable release compared to free drug	[7]
	Pharmaceutical cocrystal (DIA–β-resorcylic acid)	DIA:RA cocrystal (optimized at 1:3 molar ratio) prepared via antisolvent crystallization	Improved solubility, faster dissolution, enhanced packability/compressibility/compactibility, improved stability; novel solid phase confirmed by DSC, PXRD, FT-IR, SEM	Bioavailability ↑ 3.2-fold compared with pure DIA	Cocrystal formation modifies lattice energy and hydrogen-bonding network → enhanced solubility, improved mechanical properties, and faster dissolution → increased systemic exposure	[74]
	Eutectic-based diacerein formulations (PK evaluation study)	Novel DIA eutectics administered orally; rhein quantified using validated RP-HPLC bioanalytical method	Not directly investigated; study supports accurate PK assessment of DIA eutectics via validated quantification of rhein	PK profiling enabled: linearity r^2^ > 0.9988; validated FDA-compliant method allowed comparison of rhein exposure between pure DIA and eutectic formulations	Reliable bioanalytical quantification of rhein allows mechanistic understanding of how eutectic formulations may alter DIA absorption, metabolism, and systemic exposure	[107]
Advanced Formulations	Niosomes (ultrasonic-processed)	UP-prepared niosomes using Span 20, Pluronic L64, or mixed surfactant systems; compared with thin-film hydration (TFH)	Smaller vesicle size; more monodisperse (lower PDI); feasible entrapment; significantly faster drug release vs. pure drug and vs. TFH-niosomes	Faster release profile indicates improved dissolution and potential for enhanced bioavailability; no in vivo data reported	Reduced vesicle size + increased surface area from ultrasonic processing → accelerated drug release; improved wettability/dispersion enhances dissolution behavior of poorly soluble DCN	[42]
	Transdermal niosomes (CCD-optimized)	Niosomes prepared by thin-film hydration and optimized via central composite design; elastic vesicles developed for comparative permeation	Entrapment efficiency 95.63%; particle size 436.7 nm; PDI 0.47; zeta potential −38.8 mV; optimized lipid/surfactant system enhances vesicle stability and loading	Ex vivo: markedly enhanced skin permeation and retention vs. drug suspension; in vivo: significantly higher skin deposition (improved local availability)	Vesicular encapsulation + elastic vesicle properties enhance SC penetration; high entrapment + stable vesicle charge improves deposition, enabling efficient transdermal delivery and avoiding oral GI side effects	[117]
	Sustained-release niosomes (REV technique)	Niosomes prepared via reverse-phase evaporation using sorbitan monostearate:cholesterol ratios (5:5 to 9:1); optimized F3 (7:3)	Vesicle size 0.608–1.01 µm; PDI 0.409–0.781; entrapment 79.8% (F3); sustained release with T50% up to 10 h; zero-order kinetics (R^2^ = 0.9834); non-Fickian diffusion (*n* = 0.90)	Sustained release indicates extended drug availability at absorption site; improved dissolution performance vs. pure DCN; enhances potential oral absorption	Surfactant–cholesterol bilayers improve entrapment and modulate membrane rigidity → prolonged release; controlled diffusion reduces burst effect → more consistent systemic exposure and improved bioavailability	[118]
	Elastic bilosomes (bile salt-modified niosomes)	Bilosomes prepared by thin-film hydration with bile salts as edge activators; optimized via full factorial (B6 formulation)	Vesicle size ~301.7 nm; 100% entrapment; enhanced deformability and bilayer flexibility enabling superior penetration; improved stability vs. niosomes	Ex vivo: significantly increased permeation vs. niosomes and drug suspension. In vivo: markedly higher skin retention; histopathology confirmed dermal safety	Bile-salt-induced membrane elasticity enhances deformability → easier stratum corneum traversal; ultra-flexible vesicles increase flux + depot formation → higher local drug levels and improved therapeutic availability	[54]
	Novasomes (fatty acid-modified non-ionic vesicles)	Novasomes prepared by thin-film hydration, optimized by varying surfactant:cholesterol ratio, sonication type/time, and fatty acid level	Optimized formula (F6): vesicle size 275.2 nm, EE% 69.4%, PDI 0.309; stable vesicle architecture confirmed by FTIR and TEM	No in vivo PK reported; optimized vesicle size and EE% indicate potential for enhanced transdermal delivery and reduced GI exposure	Fatty-acid–enhanced bilayer fluidity + optimized surfactant/cholesterol balance → stable nanosized vesicles with higher loading → improved dermal penetration vs. free drug	[114]
	Elastosomes (edge-activator-based elastic vesicles)	Elastosomes optimized via 4^1^·2^1^ factorial design using different edge activators; optimal E1 formulation selected by desirability function	EE% 96.25%, PS 506 nm, PDI 0.46, ZP –38.6 mV, deformability index 12.7 g; high flexibility and stability vs. conventional vesicles	Ex vivo: significantly superior permeation + retention vs. drug suspension. In vivo: high skin deposition and confirmed dermal safety; PK: comparable systemic absorption to oral suspension; strong Level C IVIVC between release and in vivo performance	Edge activators increase bilayer elasticity → enhanced SC crossing + deeper skin deposition; high deformability + high EE% create a transdermal reservoir → improved local availability while avoiding GI exposure and oral side effects	[76]
	Hyaluosomes (HA-based gel-core vesicles for intra-articular injection)	Hyaluronic-acid gel-core vesicles optimized via full factorial design for intra-articular delivery	High entrapment efficiency 90.7%; small vesicle size ~310 nm; spherical morphology with HA core confirmed by TEM; stable zeta potential	In vivo (OA rat model): marked reduction in cartilage damage and inflammation; significant decrease in plasma TNF-α and IL-1β vs. untreated group; superior joint protection and local anti-inflammatory effect	HA-core vesicles enhance joint residence time and synovial adhesion → sustained DCN release within joint cavity; high EE% and nanosize improve drug availability at site of inflammation → leading to strong reduction in inflammatory cytokines and cartilage deterioration	[8]
	Solid Lipid Nanoparticles (SLNs)—Oral Liquid Form	Cetyl alcohol (2%) as lipid matrix; Tween 80 (0.9%) surfactant; citric acid (0.05%) for pH adjustment; structured liquid vehicle	QbD-based optimization; enhanced chemical stability of DCN in aqueous medium; protection from hydrolysis; stable for 6 months at 40 °C/75% RH; formation of uniform SLNs suitable for oral suspension (50 mg/5 mL)	Significant reduction in diarrheal side effects (*p* < 0.0001); protection of DCN from degradation; improved tolerability and safety	Entrapment within SLNs protects DCN from aqueous instability → reduces formation of rhein in colon → markedly lowers GI adverse effects; stabilization of drug within lipid matrix supports long-term storage	[126]
	Proliposomes (PLS)—Oral Controlled-Release	Film deposition method; positively charged stearylamine-containing PLS (PLS-F16); sorbitol-based PLS compressed into Eudragit RS100/Ethyl cellulose CR tablets	High EE 91.13% ± 2.25%; nano-size 128.12 ± 17.90 nm; delayed release (46.7% at 12 h); amorphization of DCN (FTIR, DSC, PXRD); improved physical stability vs. liposomes	CR tablet PT-F10 showed markedly enhanced PK: Cmax = 7455 ± 262.7 ng/mL, AUC0–24 = 913,013.7 ± 553.48 ng·h/mL, Tmax = 8 h; significantly better than marketed DCN capsules	High EE and amorphous state improved dissolution and absorption; positive charge enhanced membrane interaction; controlled-release matrix minimized colonic DCN → reduced rhein exposure and improved systemic bioavailability	[70]
	Nanostructured Lipid Carrier (NLC) Gel—Topical	DCN-loaded NLC dispersed in gel base; designed for enhanced cutaneous penetration and prolonged topical delivery	Improved rheology and spreadability; stable nano-dispersion; controlled release with extended delivery up to 24 h; optimized pH and skin compatibility; improved nanoparticle proliferation and stability	Pharmacodynamic studies confirmed faster onset and prolonged anti-inflammatory action (up to 24 h); skin irritation tests showed good dermal tolerability	Enhanced drug solubilization within lipid matrix improved penetration; nanoparticle–gel hybrid increased skin retention and sustained release, enabling prolonged analgesic/anti-inflammatory effect vs. conventional topicals	[23]
	Solid Lipid Nanoparticles (SLN) with Targeting Ligand	Chondroitin sulfate-modified DCN-loaded SLN (ChS-DC-SLN) for intra-articular targeting	Particle size 396 ± 2.7 nm; extended release up to 16 h; 2.8-fold increase in bioavailability versus plain drug; improved stability and site-specific retention	Histopathology showed improved preservation of cartilage structure; significantly higher rhein concentration at target site: 7.8 ± 1.23 µg/mL vs. 2.9 ± 0.45 µg/mL (drug dispersion); enhanced therapeutic response in OA rat model	Chondroitin sulfate acts as a homing ligand to articular cartilage → improves intra-articular accumulation; SLN matrix enhances solubility, protects DCN from degradation, and prolongs residence time → collectively increases anti-osteoarthritic efficacy	[38]
	Topical nanoemulgel (NE–Carbopol hydrogel)	Box–Behnken–optimized nanoemulsion (oleic acid 8–12%, Tween 80 10–15%, PG 15–25%) incorporated into Carbopol 934P gel	Particle size 104.3 nm; loading efficiency 18.5%; uniform drug content; 24-h release 93.6% (Higuchi, non-Fickian); good spreadability and pH stability	High in vitro permeability: cumulative permeation 12.73 μg/cm^2^ at 24 h; flux 0.574 μg/cm^2^/h; superior release and penetration vs. plain DCN	Small droplet size + oleic acid (penetration enhancer) disrupt SC lipids → increased skin permeation; hydrophilic gel network maintains sustained drug release → enhanced local therapeutic availability	[21]
	Stability-indicating analytical method (RP-HPLC) supporting nanoemulgel formulation	RP-HPLC assay developed via DoE (fractional factorial + CCD) for simultaneous quantification of DCN, ACE, and degradation products (rhein, DLS) in nanoemulgel and tablets	Validated method under ICH conditions (heat, acid/alkali, oxidative, photolytic, humidity, hydrolysis stress); high linearity (R^2^ > 0.999), accuracy 98–102%; allows reliable characterization and stability assessment of DCN nanoemulgel	Facilitates accurate quantification of DCN within novel nanoemulgel formulations; supports stability profiling and ensures dosage consistency (no direct in vivo data)	Robust analytical validation ensures precise measurement of DCN in advanced formulations → enables PK evaluation, stability assessment, and correlation of formulation performance with drug degradation behavior	[25]
	Microemulgel	Oil phase: Capmul MCM C8; Surfactant: Labrasol; Co-surfactant: Ethanol; Microemulsion incorporated into carbomer-based gel	Central Composite Design (27 runs); optimized batch F2 showed high drug solubilization capacity, thermodynamic stability, acceptable transmittance, and minimal drug–excipient interaction	In vitro diffusion showed 95% drug release within 8 h; enhanced permeation and controlled release compared to conventional topical forms	Microemulsion droplets significantly increase DCN solubilization → incorporation into gel matrix ensures controlled permeation across skin while avoiding oral GI side effects	[125]
	Microparticulate & Magnetically Responsive Delivery System	Surface-modified iron oxide microparticles (SMIOMPs) coated with chitosan for intra-articular injection	High entrapment 85.25%; particle size 1.54 µm; chitosan-coated spherical morphology; sustained drug release; stability preserved after gamma sterilization; no undesirable interactions per DSC/FTIR	Significant reduction in TNF-α, IL-1β, and knee joint diameter in arthritic rats; superior anti-inflammatory and cartilage-protective effects compared to untreated animals; improved local drug retention	Chitosan surface modification ↑ mucoadhesion + magnetic microparticles ↑ localization → enhanced intra-articular residence; sustained release moderates inflammatory mediators → improved cartilage preservation	[59]
	Stimuli-responsive polymeric hydrogel system	Rhamnogalacturonan-based linseed polysaccharide hydrogel (LSH) tablets	pH-responsive swelling/deswelling: swelling ↑ at pH 7.4; deswelling at pH 1.2; sustained release behavior; <10% release in gastric pH; SEM showed elongated porous channels; non-Fickian diffusion mechanism	Provides controlled & sustained oral release of DCN with strong gastric protection; minimizes drug hydrolysis in acidic media → potential reduction in diarrhea; enhances release consistency at intestinal pH	pH-triggered swelling at intestinal pH → enhanced diffusion; deswelling in acidic stomach → protects DCN from hydrolysis and prevents premature rhein formation; hydrogel matrix modulates diffusion kinetics	[61]
	Self-Nanoemulsifying Drug Delivery System (Solid-SNEDDS)	Rhein-loaded SNEDDS (Tween 80 + PEG 400 + Eucalyptus oil), converted into solid RS-SNEDDS	Droplet size 129 nm; zeta potential −24.6 mV; very high encapsulation (98.86%); high % transmittance (94.8%); complete amorphization confirmed by DSC/XRD; 24-h extended release (99%); smooth spherical nano-globules (FESEM)	~4-fold ↑ Cmax and ~5-fold ↑ AUC vs. free rhein; markedly enhanced oral bioavailability; significantly improved brain penetration (Cmax 2.9 µg/mL; AUC0–t 18.18 µg·h/mL)	Nanoemulsification ↓ crystallinity → ↑ solubility & dissolution; high surface area nano-droplets → enhanced lymphatic uptake; PEG/Tween system improves membrane permeability; extended-release matrix improves systemic exposure	[48]
	Mucoadhesive Oral Nanosuspension	Chitosan-coated diacerein nanosuspension (CS-DNS) prepared by sonoprecipitation	Reduced particle size; improved PDI; chitosan coating → strong positive ZP; enhanced dissolution; increased mucoadhesion; lower rhein/DCN intraluminal ratio; crystallinity retained (SEM, XRD, DSC)	Cmax ↑ to 0.74 µg/mL, delayed Tmax (3.6 h), 172% relative bioavailability vs. suspension; significantly reduced diarrheal side effect due to lower colonic rhein formation	Nanosizing ↑ surface area → improved dissolution; chitosan coat ↑ mucoadhesion → prolonged intestinal residence; controlled release ↓ colonic hydrolysis to rhein → reduces diarrhea; improved permeation in non-everted intestine explains PK improvement	[49]
	Stimuli-responsive sustained-release hydrogel	Acrylic acid/KPS/MBA cross-linked hydrogel (pH-responsive oral matrix)	pH-triggered swelling (high in pH 7.4; minimal in pH 1.2); SEM shows porous morphology; FTIR confirms crosslinking; zero-order sustained release; ↑ crosslinker → ↓ release rate (optimized M3 formulation)	Strong gastric protection; sustained intestinal release; M3 formulation showed controlled drug release ideal for chronic arthritis therapy; avoids burst release and may reduce local GI irritation	pH-sensitive swelling in basic media → promotes intestinal targeting; tighter crosslinking regulates diffusion → sustained release; acidic deswelling protects DCN from degradation and reduces conversion to rhein → potentially lowers diarrhea	[142]
	Polymeric microspheres (ionotropic gelation)	Alginate–chitosan microspheres crosslinked with CaCl_2_	Particle size 106–542 µm; ↑ chitosan → ↑ entrapment efficiency; ↑ alginate/CaCl_2_ → ↑ size; SEM: spherical & rough-surfaced microspheres; sustained release in pH 6.8; B3 (1:3 alginate:chitosan) showed slowest release	Sustained intestinal release ideal for reducing GI conversion of DCN to rhein; potential reduction in diarrhea; optimized B3 suitable for further preclinical evaluation	Ionotropic gelation → dense crosslinked matrix; higher chitosan levels ↓ hydration/porosity → prolonged release; alginate/chitosan polyelectrolyte complex regulates diffusion and protects DCN from rapid hydrolysis	[143]
	Sustained-release polymeric microspheres (in vivo efficacy evaluation)	Oral alginate–chitosan microspheres (optimized ionotropic gelation system)	Sustained-release profile confirmed in earlier formulation paper; controlled intestinal release; slower transit & prolonged retention	In CFA-induced arthritis: reduced paw edema, improved gait score, lower arthritic index, reduced joint stiffness, radiographic improvement; therapeutic effect comparable to free diacerein API and glucosamine; charcoal meal test showed slower GI transit → reduced diarrhea	Sustained release → reduced colonic conversion of DCN to rhein → lower diarrheal side effect; prolonged systemic exposure → improved anti-inflammatory response (IL-1β pathway), matching efficacy of standard treatments	[144]
	Microneedle-assisted percutaneous system	Self-dissolving microneedles + CMC-based gel containing PEG4000 solid dispersion of diacerein	PEG400 & PEG4000 solid dispersion → markedly ↑ solubility & amorphization (confirmed by XRD); microneedles dissolved in 5 min; uniform needle morphology; microchannel formation validated; ↑ MN loading (390 µg/array); optimized gel enabled 98% permeation	2.43-fold ↑ permeation, 74.39% ex vivo permeation via MN-array; 15.75% skin deposition; significantly improved anti-inflammatory activity in paw-edema model; reduced diarrheal episodes vs. oral marketed formulation; stable under accelerated conditions	Solid dispersion ↓ crystallinity → ↑ solubility; dissolving MNs bypass stratum corneum → enhanced percutaneous flux; microchannels + CMC gel create sustained depot; reduced GI exposure ↓ colonic hydrolysis → ↓ diarrhea	[147]
	Non-formulation pharmacological adjuvant study	Co-administration of diacerein (100 mg/kg) with methanolic *Adenium obesum* extract	No formulation modification; systemic administration of pure DCN	Significant neuroprotective effect in Parkinsonism & depression models: improved motor coordination (rotarod), reduced depressive behavior (forced swim), improved exploratory behavior (hole-board); histopathology showed neuroprotection; antioxidant markers improved (↑ GSH, SOD, CAT; ↓ LPO)	DCN’s IL-1β inhibitory and anti-inflammatory properties may synergize with phytochemicals of A. obesum; reduction in neuroinflammation supports rationale for using DCN as a broad-spectrum anti-inflammatory candidate in advanced formulations	[2]
	Transdermal hydrogel–nanoemulsion co-delivery system	Xanthan hydrogel containing nanoemulsion-loaded diacerein + glucosamine sulfate	Nanoemulsion with globule size 81.95 nm; PDI 0.285; ZP +39.3 mV → high stability; thixotropic rheology; uniform morphology (CryoSEM/TEM); controlled non-Fickian release; stable under ICH conditions; good skin biocompatibility	Significant reduction in TNF-α, CRP, HMGB1, MCP-1; strong chondroprotection in OA rat model; reduced inflammation & cartilage degradation; controlled permeation across skin	Small droplet size ↑ penetration; xanthan matrix sustains release; synergy of glucosamine (chondroprotective) + DCN (IL-1β inhibitor) → amplified anti-inflammatory & cartilage-protective effect; positive ZP enhances stability & interaction with skin	[55]
	Topical transferosomal hydrogel (dual-drug system)	1% hyaluronic acid gel containing transferosomes co-loaded with berberine HCl + diacerein	Homogeneous gel, pH 5.4 ± 0.4; pseudoplastic rheology; spreadability 9.8 g·cm/s; sustained 24 h release (DCN: 88.44%, BBR: 81.56%); improved lipid vesicle penetration & residence	Significant reduction in erythema, scaling, and epidermal thickness; reduced acanthosis in histology; marked decrease in TNF-α and IL-17A levels in psoriatic mice; improved local therapeutic effect	Transferosomes enhance deep dermal penetration; HA gel increases hydration & skin residence; combination of berberine (anti-inflammatory/anti-proliferative) with diacerein (IL-1β inhibitor) provides synergistic suppression of psoriatic cytokines & keratinocyte hyperproliferation	[148]
	Dual-drug transferosomal nanocarriers (optimized via Box–Behnken design)	Film-hydration transferosomes containing berberine HCl + diacerein; sodium deoxycholate as edge activator	Particle size 110.90 ± 2.8 nm; PDI 0.296; ζ = −13.3 mV; high EE% (BBR 89.50 ± 1.5%, DCN 91.23 ± 1.8%); deformability index 2.44; 24 h sustained release (BBR 82.09%, DCN 85.02%); antioxidant activity 38.36%; stable at 4 °C and 25 ± 2 °C/60 ± 5% RH for 3 months	Enhanced transdermal flux (BBR 0.0224 μg cm^−2^ h^−1^; DCN 0.0462 μg cm^−2^ h^−1^); Raman mapping confirmed dermal penetration; no irritation in *BALB/c* mice	Sodium–deoxycholate-based transferosomes improve membrane elasticity and deep skin permeation; co-delivery of two cytokine-modulating agents supports multi-pathway suppression of psoriatic inflammation (TNF-α, IL-12, IL-23)	[149]
	Dual-drug targeted solid lipid nanoparticles (SLNs) containing Rhein + Methotrexate	Solid lipid nanoparticles decorated with RH and MTX for targeted RA delivery	Nanosized particles; high negative zeta potential → high stability (exact size not stated numerically but reported as “suitable nanosized range”); good physicochemical attributes	Significant improvement in inflammatory and arthritic markers in vivo; improved joint histology and ultrastructure; reduced disease progression in adjuvant arthritis model	SLNs altered endoplasmic-reticulum-stress (ERS)–mediated apoptosis—suggesting mechanistic modulation of RA pathways; improved delivery enhances anti-inflammatory efficacy of RH despite low inherent bioavailability	[150]

Abbreviations: SD: Solid Dispersion, PEG: Polyethylene Glycol, HPMC: Hydroxypropyl Methylcellulose, PVP: Polyvinylpyrrolidone, QbD: Quality by Design, DSC: Differential Scanning Calorimetry, XRD: X-ray Diffraction, SEM: Scanning Electron Microscopy, PBPK: Physiologically Based Pharmacokinetic, ODTs: Orally Disintegrating Tablets, β-CD: Beta-Cyclodextrin, HP-β-CD: Hydroxypropyl Beta-Cyclodextrin, FTIR: Fourier Transform Infrared Spectroscopy, PXRD: Powder X-ray Diffraction, RA: Resorcylic Acid, RP-HPLC: Reverse Phase High-Performance Liquid Chromatography, UP: Ultrasonic Processing, TFH: Thin Film Hydration, CCD: Central Composite Design, PDI: Polydispersity Index, ZP: Zeta Potential, SC: Stratum Corneum, REV: Reverse-Phase Evaporation, EE%: Entrapment Efficiency, HA: Hyaluronic Acid, SLNs: Solid Lipid Nanoparticles, RH: Rhein, MTX: Methotrexate, ICH: International Council for Harmonisation, CR: Controlled Release, NLC: Nanostructured Lipid Carrier, NE: Nanoemulsion, PLS: Proliposomes, FESEM: Field Emission Scanning Electron Microscopy, API: Active Pharmaceutical Ingredient, CMC: Carboxymethyl Cellulose, CS-DNS: Chitosan-Coated Diacerein Nanosuspension, GSH: Glutathione, SOD: Superoxide Dismutase, CAT: Catalase, LPO: Lipid Peroxidation, CRP: C-Reactive Protein, HMGB1: High Mobility Group Box 1, MCP-1: Monocyte Chemoattractant Protein-1, TNF-α: Tumor Necrosis Factor Alpha, IL-1β: Interleukin-1 Beta, IL-17A: Interleukin-17A, IL-12: Interleukin-12, IL-23: Interleukin-23, ERS: Endoplasmic Reticulum Stress.

## Data Availability

No new data were created or analyzed in this study.

## Abbreviations

AA: Acrylic Acid; AI: Artificial Intelligence; ANN: Artificial Neural Network; API: Active Pharmaceutical Ingredient; AUC: Area Under the Curve; AUC_0–6h_: Area Under the Curve from 0 to 6 h; BCS: Biopharmaceutics Classification System; β-CD: Beta-Cyclodextrin; Bet6: Betaine-Based Ionic Liquid; Carn6: L-Carnitine-Based Ionic Liquid; Carbopol 934/940: Polyacrylic Acid-Based Gelling Agent; CAT: Catalase; CCD: Central Composite Design; CD: Cyclodextrin; CD-MOFs: Cyclodextrin-Based Metal–Organic Frameworks; ChS: Chondroitin Sulfate; ChS-DC-SLNs: Chondroitin Sulfate-Modified Diacerein-Loaded Solid Lipid Nanoparticles; Cmax: Maximum Plasma Concentration; CMC: Carboxymethyl Cellulose; COX: Cyclooxygenase; COVID-19: Coronavirus Disease 2019; CR: Controlled Release; CRP/Hs-CRP: (High-sensitivity) C-Reactive Protein; CS-DNS: Chitosan-Coated Diacerein Nanosuspension; DCN: Diacerein; DI: Deformability Index; DM: Diabetes Mellitus; DHA: 2,4-Dihydroxybenzoic Acid; DSC: Differential Scanning Calorimetry; EB/EBS: (Simplex) Epidermolysis Bullosa; EE/EE%: Entrapment Efficiency; EMA: European Medicines Agency; ERS: Endoplasmic Reticulum Stress; Ethyl Cellulose: Hydrophobic Polymer for Controlled Release; Ex vivo: Outside a Living Organism; Ethanol/Propylene Glycol/Transcutol-P: Penetration Enhancers/Co-surfactants; ExPreSo: Excipient Prediction Software; FDA/USFDA: United States Food and Drug Administration; FDT/ODTs: (Orally) Fast-Dissolving Tablets; FESEM: Field Emission Scanning Electron Microscopy; FMA: Fumaric Acid; FTIR: Fourier Transform Infrared Spectroscopy; GI: Gastrointestinal; GR/IR/SR: Gastro-Retentive/Immediate-Release/Sustained-Release; GSH: Glutathione; HA: Hyaluronic Acid; HCl: Hydrochloride; Higuchi Model: Drug Release Kinetic Model; HMGB1: High Mobility Group Box 1; HPH: High-Pressure Homogenization; HPLC/RP-HPLC: (Reversed-Phase) High-Performance Liquid Chromatography; HPMC: Hydroxypropyl Methylcellulose; HPβCD/HP-β-CD: Hydroxypropyl-Beta-Cyclodextrin; ICH: International Council for Harmonisation; IL/ILs: Ionic Liquids/Interleukins (Context-dependent); IL-1β/IL-12/IL-17A/IL-23: Interleukins 1β, 12, 17A, 23; In vivo: Within a Living Organism; Intra-articular: Injection into Joint Space; IR: Immediate-Release; ITP: Immune Thrombocytopenic Purpura; Labrasol/Kolliphor EL/Tween 80: Nonionic Surfactants; Lecithin–Cholesterol: Liposomal Stabilizing Agents; LPO: Lipid Peroxidation; MAFLD: Metabolic Dysfunction-Associated Steatotic Liver Disease; MCP-1: Monocyte Chemoattractant Protein-1; MDT: Mean Dissolution Time; min/h/°C: Minute/Hour/Degree Celsius; ML: Machine Learning; MTX: Methotrexate; mV: Millivolt; µg/mL: Micrograms per Milliliter; µg/cm^2^/h: Micrograms per Square Centimeter per Hour; µm/nm: Micrometer/Nanometer; NA: Not Applicable; NCT: National Clinical Trial Identifier; NE: Nanoemulsion; NLC/NLCs: Nanostructured Lipid Carrier(s); NSAIDs: Nonsteroidal Anti-inflammatory Drugs; OA/RA: Osteoarthritis/Rheumatoid Arthritis; PBPK: Physiologically Based Pharmacokinetic; PDI: Polydispersity Index; PEG: Polyethylene Glycol; PEO: Polyethylene Oxide; PHASE 1/2/3/4: Phases of Clinical Trials; pH: Potential of Hydrogen; PLGA: Poly(lactic-co-glycolic acid); PLS: Proliposomes; PVP: Polyvinylpyrrolidone; PXRD/XRD: (Powder) X-ray Diffraction; QbD: Quality by Design; RA (contextual): Resorcylic Acid; RH: Rhein; ROS: Reactive Oxygen Species; SC: Stratum Corneum; SD/SDs: Solid Dispersion(s); SDS: Sodium Dodecyl Sulfate; SEDDS/SNEDDS: (Self-)Emulsifying/Nanoemulsifying Drug Delivery Systems; SEM: Scanning Electron Microscopy; SLN/SLNs: Solid Lipid Nanoparticle(s); SOD: Superoxide Dismutase; SR: Sustained Release; SVM: Support Vector Machine; SYSADOAs: Symptomatic Slow-Acting Drugs for Osteoarthritis; T50%: Time to Release 50% of the Drug; TFH/THF: Thin-Film Hydration; Tmax: Time to Reach Cmax; TNF-α: Tumor Necrosis Factor-Alpha; Topical: Application to Skin or Mucosa; Transcutol-P/Ethanol/Propylene Glycol: Penetration Enhancers/Co-surfactants; UP: Ultrasonic Processing; *w*/*w*/%: Weight by Weight/Percent; ZP: Zeta Potential.
